# Redox compromise as an early, modifiable entry point in schizophrenia: a staging framework for early intervention

**DOI:** 10.3389/fnins.2026.1941148

**Published:** 2026-10-15

**Authors:** Leonard A. Lado, Aruna Misir

**Affiliations:** 1 Lado Healing Institute, Bonita Springs, FL, United States; 2 Windsor University School of Medicine, Cayon, Saint Kitts and Nevis

**Keywords:** biomarkers, clinical staging, early intervention, glutathione, lithium, neuroprogression, oxidative stress, schizophrenia

## Abstract

Schizophrenia is understood as a disorder of dopamine signaling and is treated with dopamine receptor antagonists. These drugs reduce hallucinations and delusions but leave cognitive impairment, negative symptoms and functional decline largely untouched. This article advances a complementary view, as a hypothesis rather than an established account. It treats redox compromise, meaning chronic oxidative stress, glutathione depletion, mitochondrial inefficiency and the inflammatory state that accompanies them, as an early and modifiable point of entry into a self-reinforcing loop. The loop also involves NMDA receptor hypofunction, impairment of parvalbumin interneurons, disturbed circuit connectivity and, last, the dopaminergic phenotype that current drugs address. It runs in both directions: blocking NMDA receptors itself generates oxidative stress in animal models, so the claim is not that redox compromise comes first, but that it can be measured and modified early, before the changes that current treatment cannot reverse. The clinical staging framework of McGorry and the neuroprogression framework of Berk provide the architecture for translating this into intervention windows, with the first two levels treated as risk strata rather than disease stages. Impaired glucose tolerance in first-episode patients who have never taken antipsychotics, which neither medication nor chronicity can explain, is presented as evidence that metabolic disturbance is a parallel expression of the same systemic process. The progressive framing is contested. The principal counterevidence, including the contribution of antipsychotic exposure to brain volume loss and the largely stable course of cognitive impairment after onset, is set out together with the results that would require abandoning it. We organize the biomarker literature by stage and propose three predictions that a trial could refute: that biomarker-stratified prevention will outperform unselected prevention at the clinical high-risk stage; that medial prefrontal glutathione and mismatch negativity deficit will add predictive value beyond clinical assessment; and that removing the redox measures from a multimodal panel will worsen its prediction of transition. A biomarker-stratified phase 2b trial with a formal power calculation is proposed. The human evidence supports association and biological plausibility rather than proven causal direction.

## 1 Introduction

Schizophrenia has long been treated as a fixed disorder of brain chemistry, managed by blocking dopamine to quiet its most visible symptoms. This manuscript proposes a different picture. We argue that schizophrenia may be better understood as a process that begins years before psychosis, with a measurable imbalance in the brain’s handling of oxidative stress and inflammation. In this view, the dopamine disturbance that current drugs target is not the root cause but a later expression of a loop into which redox compromise offers an early point of entry. If this is correct, three things follow. First, the process can be staged, from silent risk through early warning signs to full illness. Second, the upstream biology is measurable through biomarkers and, in its early stages, at least partly reversible. And third, intervening early, while the biology is still drifting rather than fixed, offers a chance to change the illness’s course rather than only manage its symptoms.

The manuscript builds this case in a deliberate order. It first contrasts the prevailing downstream, dopamine-centered model with the upstream view, establishing why the distinction matters (Section 2). It then examines the upstream biology itself, showing how oxidative stress acts as an integrative hub that connects genetic vulnerability, inflammation, mitochondrial dysfunction and environmental insult, and how that convergence damages the specific cells and circuits implicated in the disease, and how the biomarker evidence for each component of that node stands up to scrutiny (Section 3). From this foundation it constructs the practical architecture: a clinical framework spanning two risk strata and three stages, with defined intervention windows (Section 4), and a review of the therapies that act on upstream biology and when they are most likely to help (Section 5). Finally, and most importantly, it puts the entire framework at risk by translating it into three explicit, falsifiable predictions and a concrete trial design that could confirm or refute them (Section 6), before weighing what the framework does and does not establish (Section 7).

The intended utility of this framework is practical as much as conceptual. For research, it offers a common staging language and a subtyping scheme that could make trials more informative: rather than testing one intervention against an undifferentiated high-risk group, investigators could stratify by underlying biology, inflammatory or redox, and match each subgroup to a mechanism-appropriate treatment, an approach that may rescue signals lost in unstratified designs. It also supplies falsifiable hypotheses and a ready trial architecture. Should the framework be validated, the clinical applications are more consequential: earlier biomarker-guided identification of those most likely to progress, treatment matched to biological subtype rather than diagnosis alone, and a rationale for upstream-acting interventions during the window when biology is still modifiable. In the nearer term, the staging structure provides an organized way to think about where a patient sits on the trajectory and what is justified at each stage. These applications are prospective. The framework is a research program to be tested, not a clinical protocol to be adopted.

Schizophrenia affects approximately 24 million people worldwide and remains one of the leading causes of years lived with disability (Charlson et al., 2018; Kahn et al., 2015; Owen et al., 2016). Despite the substantial burden, two features of the clinical landscape have remained stubbornly resistant to change. First, no biomarker has reached the threshold for routine diagnostic use; diagnosis continues to depend on syndromic criteria (Sierakowska et al., 2025; Yue et al., 2022). Second, the therapeutic armamentarium continues to be dominated by D2 receptor antagonism, an approach which addresses positive symptoms but leaves cognitive impairment, negative symptoms and progressive functional decline largely untouched (Kahn et al., 2015; McCutcheon et al., 2020). The dominant therapeutic paradigm intervenes downstream at the point of the dopamine signal, rather than upstream, at the level of the biology that produces the dopamine signal in the first place.

A complementary view has matured over the past two decades. It treats schizophrenia not as a static neurotransmitter disorder but as a disorder whose trajectory unfolds across identifiable stages. In this view, articulated principally by McGorry and colleagues (McGorry et al., 2006; McGorry et al., 2018; McGorry and Hickie, 2019), psychotic illness moves from asymptomatic risk through non-specific symptoms, attenuated psychotic symptoms, first episode and chronic illness, with the biological substrate becoming progressively more consolidated and less reversible at each stage. The parallel neuroprogression framework, articulated by Berk and colleagues (Berk et al., 2011; Davis et al., 2016), specifies the upstream biology that drives this progression: oxidative stress, mitochondrial dysfunction, neuroinflammation, neurotrophin loss and impaired neuroplasticity. The biomarker literature, which has matured from single-analyte hypothesis-testing to multi-omic integration (Lozupone et al., 2017; Zaki et al., 2022), now provides increasingly specific tools for measuring this upstream biology and tracking it across stages.

These three traditions have rarely been integrated explicitly. The integration we propose makes a single substantive claim: that redox and inflammatory imbalance is an early and modifiable point of entry into a self-reinforcing loop whose downstream expression includes the well-validated dopaminergic abnormality, rather than a late consequence of that abnormality. If this framing is correct, two implications follow. First, the timing of intervention matters in a biologically grounded way: earlier intervention targets biology that is still drifting and at least partially reversible, whereas later intervention targets biology that has consolidated. Second, the choice of intervention should reflect upstream biology where possible: low-dose lithium, N-acetylcysteine, omega-3 fatty acids and other redox-active agents act on the upstream node, whereas D2 antagonism acts on the downstream phenotype.

The clinical staging structure originates with McGorry; the neuroprogression formulation originates with Berk; the biomarker findings are drawn from a literature ably synthesized elsewhere (Lozupone et al., 2017; Sierakowska et al., 2025; Zaki et al., 2022; Yue et al., 2022). The contribution of this manuscript is to bring these into a single framework that yields specific, testable predictions and a concrete trial design that the field’s center of gravity should shift from downstream symptom management to upstream disease modification.

### 1.1 A note on motivation and on impaired homeostasis as a unifying principle

The motivation for the present work is partly clinical. A patient maintained on long-term lithium reached his ninety-fifth year with conspicuously preserved health and a low burden of the comorbidities that ordinarily accumulate in late life. Set against the literature on lithium’s effects on glycogen synthase kinase-3, neurotrophic signaling, mitochondrial function, autophagy and antioxidant defense (Berger et al., 2012; Chiu et al., 2013; Ghanaatfar et al., 2023), that observation prompted a longer inquiry into what the pattern across diseases might be. What emerged, across neurodegeneration, psychiatric illness and chronic systemic disease, is that oxidative stress and impaired redox homeostasis recur as upstream contributors, often years or decades before the syndrome that names the disease appears.

The present framework extends to schizophrenia the homeostasis-centered model we developed in prior work (Lado and Misir, 2026), which argued that impaired homeostasis is a fundamental principle of illness too easily obscured when attention is focused on diagnostic detail. Homeostasis is the precondition that mechanism-by-mechanism enumeration of pathology can describe but not on its own restore; the further one moves from intact homeostasis, the more limited the value of downstream symptomatic intervention is likely to be.

Central to redox homeostasis is a layered set of endogenous antioxidant systems whose biology is well characterized but whose clinical implications remain under-studied in psychiatric disease. The brain maintains a constitutive defense comprising enzymatic components (superoxide dismutase, catalase, glutathione peroxidase, peroxiredoxins, thioredoxin reductase), non-enzymatic scavengers (glutathione, the major intracellular thiol antioxidant, alongside ascorbate, urate and α-tocopherol), and a transcriptional response centered on the Nrf2–Keap1 axis, which inducibly upregulates more than two hundred cytoprotective genes when redox tone shifts toward pro-oxidant conditions (Halliwell, 2006; Halliwell and Gutteridge, 2015; Dringen, 2000; Hayes and Dinkova-Kostova, 2014).

The Nrf2–Keap1 axis is the cell’s master switch for antioxidant defense. Nrf2 is a transcription factor that turns on protective genes; Keap1 is its sensor and brake, normally holding Nrf2 inactive and marking it for degradation. When oxidative stress rises, Keap1 releases its grip and a broad set of antioxidant and detoxification genes is switched on. Its setpoint and responsiveness can be weakened by genetic variation or chronic stress, a point that recurs throughout this paper. Sies and Jones (2020) distinguish physiological oxidative eustress, in which low-level reactive oxygen species act as signaling molecules and recruit Nrf2-mediated adaptation, from oxidative distress, in which production overwhelms defense and damage accumulates. Figure 1 summarizes this architecture. What remains to be understood is how these mechanisms function in psychiatric illness, how genetic variation modulates their capacity, and how chronic upstream pressure (from inflammation, mitochondrial dysfunction, kynurenine pathway activation, or environmental insult) converts eustress into distress.

**Figure 1:**
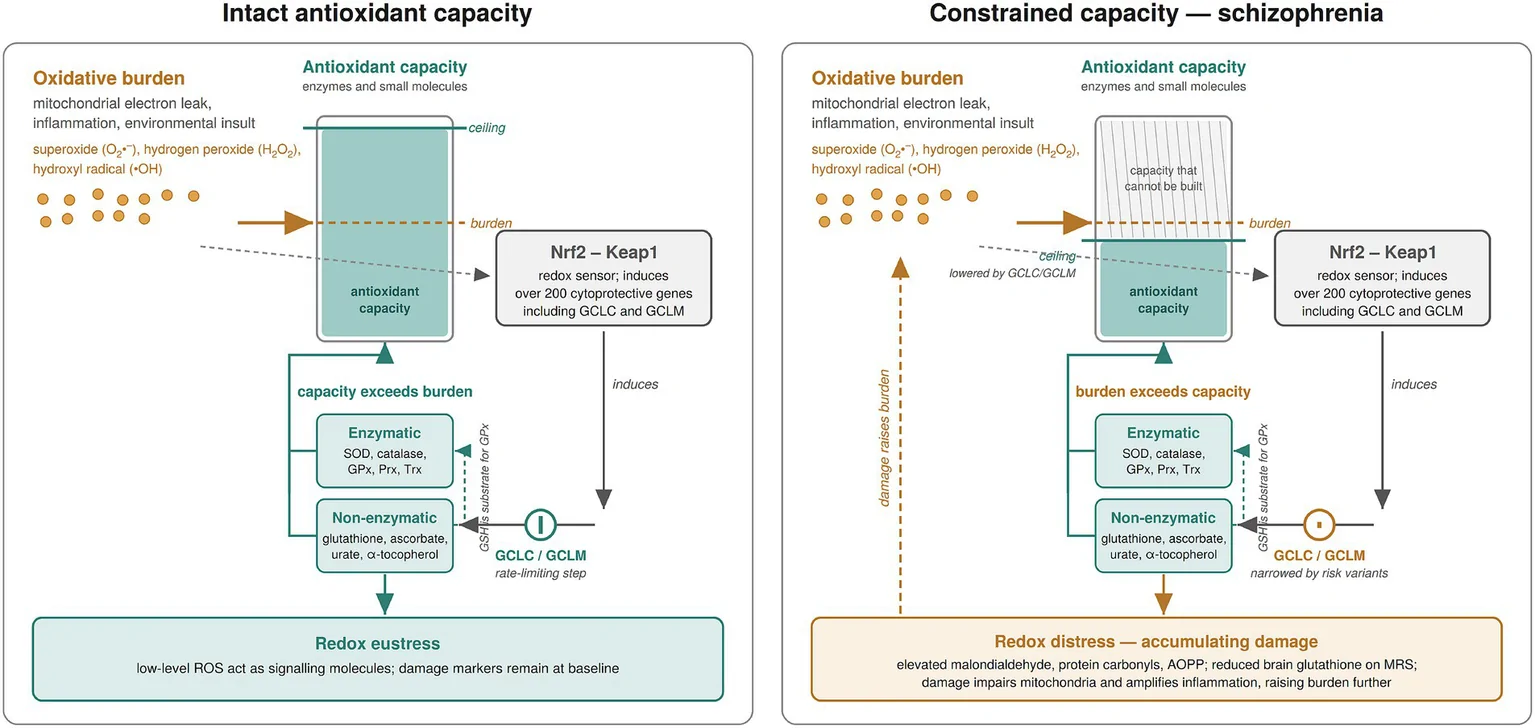
Antioxidant capacity and the constraint imposed by reduced glutathione synthesis. Both panels use the same scale and the same oxidative burden, so capacity is the only variable. Amber circles are reactive oxygen species; the dashed line is the burden requiring neutralization; the shaded column is the capacity available to meet it; the horizontal bar is the ceiling on how much capacity can be built. Left: Nrf2-Keap1 senses oxidation and induces the enzymatic and non-enzymatic pools, including the glutathione-synthesis genes GCLC and GCLM, which encode the catalytic and modifier subunits of glutamate-cysteine ligase, and capacity rises above burden. Right: risk variants narrow that rate-limiting step, lowering the ceiling before any insult arrives. Because glutathione is also the substrate for glutathione peroxidase, the constraint reduces the enzymatic arm’s effective activity as well as the non-enzymatic pool. The hatched region is capacity that cannot be built rather than capacity consumed; antioxidant defense is reduced in schizophrenia, not absent. The same burden now exceeds capacity, and the resulting damage impairs mitochondria and amplifies inflammation, raising burden further.

The schizophrenia literature is consistent with progressive failure of these defenses; Section 3 treats the evidence in full, and a brief summary orients the reader here. Patients show reduced brain glutathione on magnetic resonance spectroscopy, altered antioxidant enzyme activities, and elevated peripheral markers of oxidative damage; variation in the rate-limiting glutathione-synthesis enzymes GCLC and GCLM is associated with the disorder and with brain-structural patterns consistent with reduced antioxidant capacity (Das et al., 2019; Flatow et al., 2013; Gysin et al., 2007; Tosic et al., 2006; Puglisi-Allegra et al., 2021). What emerges is a defense system sufficient under ordinary conditions but inadequate under sustained insult, and which once inadequate contributes to the mitochondrial, inflammatory and excitotoxic perturbations that worsen it further. Disrupted homeostasis and impaired antioxidant machinery are two descriptions of one process at different scales. Restoring that machinery, whether pharmacologically, nutritionally, or by reducing upstream load, is therefore an upstream target rather than a peripheral adjunct, and one the staging framework below is designed to make operationally addressable.

This manuscript advances, as one of its central propositions, that environmental insults including childhood trauma engage oxidative stress as an intermediate mechanism, and that the resulting redox imbalance reshapes the epigenetic expression of schizophrenia-relevant genes, providing a biochemical pathway through which gene–environment interaction translates risk into illness. The evidence bearing on this proposition, and the limits of what it currently supports, are set out in Section 3.4.

The purpose of the present model is threefold. First, to make visible a disease trajectory whose apparent inevitability has, we believe, been overestimated: if oxidative stress and impaired homeostasis are genuine upstream contributors, the disease is not as fatal in its biology as it appears in its late clinical picture, and biomarker-guided early intervention becomes a falsifiable research direction rather than a wishful one. Second, to give clinicians and researchers a research framework: a staging structure with biomarker subtypes and stage-proportionate intervention windows that can be tested in real cohorts and refined or rejected on the evidence. Third, to give families a defensible reason for hope. The research strategy borrows from oncology, where outcomes changed through staging, biomarker-guided stratification and early intervention rather than a single breakthrough; the borrowing is methodological only.

### 1.2 Evidence base and how it was assembled

This is a Hypothesis and Theory article, not a systematic review. The evidence gathered here was chosen to test whether a proposed framework holds together, not to survey the field completely. Sources were found through PubMed and Google Scholar, using terms covering oxidative stress, redox regulation, glutathione, neuroinflammation, clinical staging and neuroprogression in schizophrenia and psychosis. Where meta-analyses, longitudinal cohorts or randomized trials were available, these were given priority.

The argument draws on evidence of very different strength. Three tiers are therefore distinguished, and are used consistently throughout.

*Established* findings are supported by meta-analysis, or by several independent human studies pointing the same way. Reduced glutathione in the brain in first-episode patients, raised inflammatory markers in blood, and reduced mismatch negativity all belong here.

*Model-supported* findings come from experiments in animals or cells, where the variable of interest was directly manipulated, but have not been shown in humans. The proposed sequence from glutathione depletion to parvalbumin interneuron injury to disrupted gamma activity rests mainly on this tier. The framework’s causal ordering depends on it.

*Extrapolated* findings are those where a mechanism shown in one setting is applied to schizophrenia by inference. The proposed relationship between redox state and clinical stage is extrapolated in this sense. It is a hypothesis, not an established relationship.

Supplementary Table S1 lists, for every body of evidence cited in Sections 3, 4, the study design, illness stage, treatment status, whether the marker was measured centrally or peripherally, the direction of the finding, and its main limitation. Findings that were null, or that pointed the other way, are included rather than left out. For example, a recent study of 521 patients who had never taken antipsychotics found that higher serum uric acid went with worse cognition, not better, which runs against the antioxidant-capacity interpretation (Zhang et al., 2026).

## 2 Upstream versus downstream: a reframing of the disease

The dopamine hypothesis remains the most pharmacologically validated framework in schizophrenia research. Presynaptic striatal dopamine synthesis capacity, measured by [18F]-DOPA PET, is the most consistent neurochemical abnormality and predicts transition to psychosis in clinical high-risk (CHR) individuals, also referred to as ultra-high-risk (UHR) in the Melbourne early-psychosis tradition, defined by attenuated psychotic symptoms, brief limited intermittent psychotic symptoms, or genetic risk plus functional decline (Howes and Kapur, 2009; McCutcheon et al., 2019; Fusar-Poli et al., 2013a; Yung and Nelson, 2011). The pharmacological corollary, that antipsychotic efficacy depends on D2 antagonism, dominated treatment for more than six decades, and the recent approval of xanomeline-trospium establishes that D2 antagonism is not necessary for antipsychotic efficacy. It does not establish that dopaminergic signaling is bypassed, since M4 muscarinic agonism acts in part by modulating striatal dopamine release presynaptically. But the dopamine hypothesis answers a narrower question than it is sometimes credited with: it explains how positive symptoms are generated and how D2 antagonism abolishes them. It does not explain why the dopaminergic abnormality arises, why cognitive impairment is present before psychotic symptoms emerge, or why the disease has the progressive course that the staging literature has documented (Kahn et al., 2015; McCutcheon et al., 2020; Murray et al., 2017).

A complementary set of frameworks addresses the upstream biology producing the dopaminergic abnormality. The neurodevelopmental hypothesis situates schizophrenia in altered cortical development driven by genetic vulnerability interacting with environmental insults during sensitive periods (Birnbaum and Weinberger, 2017; Murray et al., 2017). The NMDA hypofunction hypothesis, supported by the psychotomimetic action of ketamine and PCP and by post-synaptic density genetics, places glutamatergic dysregulation upstream of dopaminergic disinhibition (Coyle, 2012; McCutcheon et al., 2020). The parvalbumin interneuron hypothesis, developed principally by the Lewis and Do groups, identifies fast-spiking GABAergic interneurons as the cellular substrate whose impairment links NMDA hypofunction to cortical gamma-band abnormalities and cognitive dysfunction (Cabungcal et al., 2013; Gonzalez-Burgos and Lewis, 2012; Steullet et al., 2017). These interneurons express the calcium-binding protein parvalbumin, fire rapidly, and act as pacemakers that synchronize surrounding excitatory neurons to produce the gamma rhythms supporting working memory, attention and cortical integration. They are, in effect, where upstream redox injury is converted into downstream circuit dysfunction.

Two observations tie these upstream processes together. First, the cellular endpoint on which they converge, impairment of parvalbumin interneurons, is exquisitely sensitive to oxidative injury. These cells have high metabolic demand from fast-spiking activity, are wrapped in redox-sensitive perineuronal nets, and have limited regenerative capacity (Cabungcal et al., 2013; Steullet et al., 2017). Animal models in which oxidative stress is induced, for example through reduced glutathione synthesis, produce parvalbumin interneuron loss and schizophrenia-consistent behavioral phenotypes, at least partly reversed by N-acetylcysteine during sensitive developmental windows (Cabungcal et al., 2013; Cabungcal et al., 2014). Second, the redox compromise is measurable in patients. Medial prefrontal glutathione is reduced on magnetic resonance spectroscopy: the original *in vivo* report described a 52% reduction in drug-free patients (Do et al., 2000), while subsequent meta-analyses of larger pooled samples report a smaller but consistent effect (Das et al., 2019; Sydnor and Roalf, 2020). Peripheral oxidative damage markers (malondialdehyde, advanced oxidation protein products and protein carbonyls) are reliably elevated (Ben Othmen et al., 2008; Flatow et al., 2013; Juchnowicz et al., 2021).

This convergence supports a reframing of schizophrenia in which an upstream redox imbalance, embedded in a network of mitochondrial inefficiency, neuroinflammation and impaired neuroplasticity, is positioned early in the process that generates the cellular, circuit and neurotransmitter abnormalities producing clinical symptoms. The ordering is the hypothesis under test rather than an established fact, and the animal evidence supports both directions: repeated NMDA-receptor antagonism raises neuronal superoxide through NADPH oxidase and produces the same parvalbumin interneuron phenotype (Behrens et al., 2007), so redox compromise can lie downstream of NMDA hypofunction as readily as upstream of it. Section 3.7 sets out that evidence and the loop formulation it supports; Section 3.8 sets out the case against describing the illness as progressive at all. Most of the human evidence assembled here is cross-sectional, and establishes association and biological plausibility rather than proven causal direction; the strongest causal evidence comes from animal models in which induced oxidative stress produces schizophrenia-relevant deficits that are reversed by antioxidant treatment during sensitive windows (Cabungcal et al., 2013; Cabungcal et al., 2014). The directional language throughout is the hypothesis under test, not an established fact. Figure 2 illustrates the integration of three traditions (clinical staging, neuroprogression, biomarker evidence) into this upstream-downstream framework.

**Figure 2:**
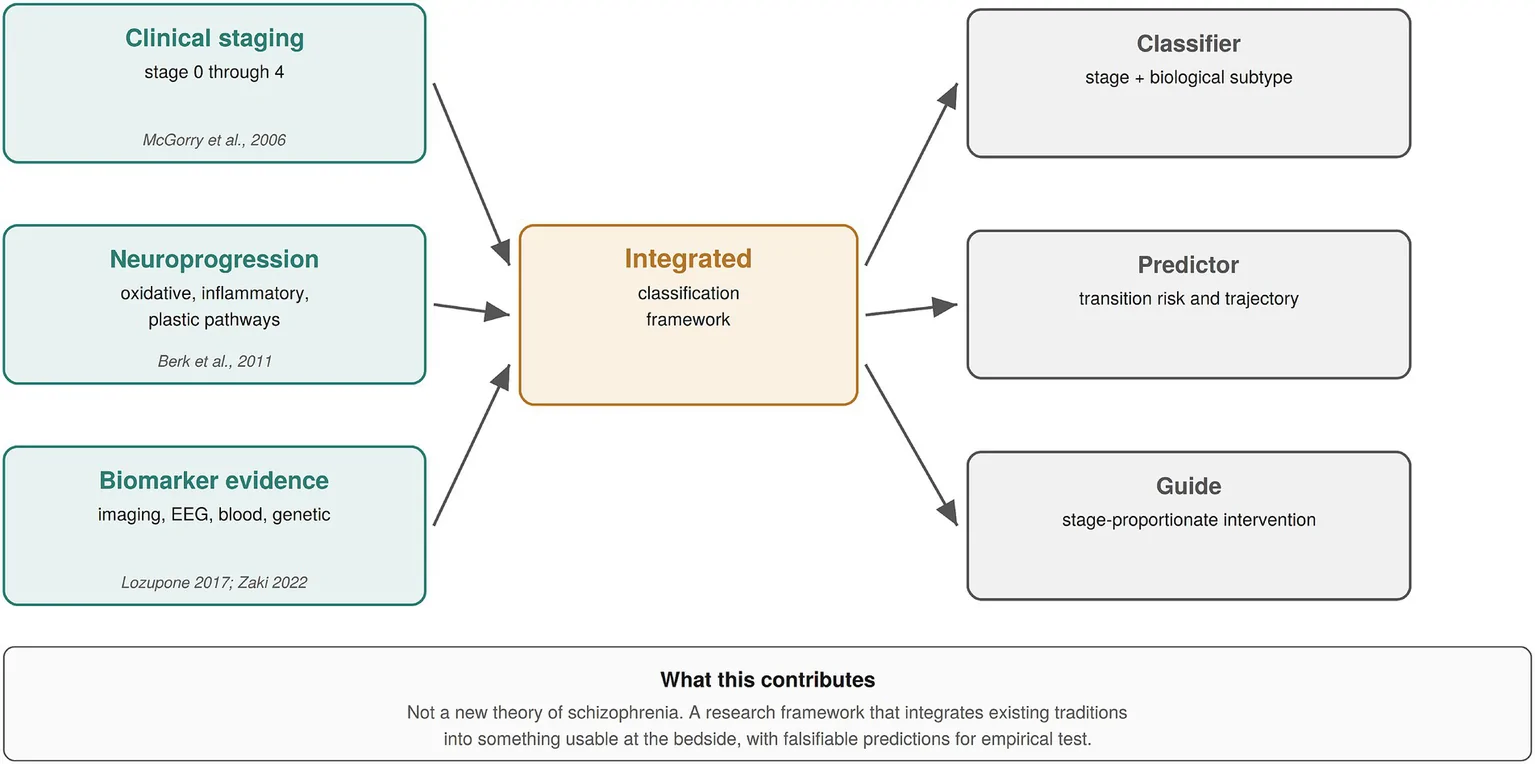
Three traditions converge into a classification framework.

Recent evidence bears on whether the late-stage cognitive trajectory is intrinsic to the disease or an incidental comorbidity. A recent study of patients with severe, treatment-resistant schizophrenia examined the dementia that can develop late in the illness (Pathak et al., 2026). This dementia looked like the cognitive impairment of ordinary schizophrenia, only more advanced, and it could not be explained by Alzheimer disease, by other neurodegenerative disorders, by antipsychotic medication, or by cardiovascular illness. In other words, the late-stage decline appears to come from schizophrenia itself, as the same disease process carried further, rather than from a separate condition added on top. This is consistent with a progressive account in the subgroup with severe, treatment-resistant illness, though it does not establish one for the illness as a whole (Section 3.8). Notably, that study described this endpoint but did not identify its cause, which the present framework proposes may be redox-related and most modifiable early.

Two clarifications are worth making. First, the upstream-downstream framing does not deny the dopaminergic abnormality; it situates it as a downstream consequence of upstream biology. D2 antagonism remains effective for positive symptoms and is unlikely to be displaced as the first-line treatment for first-episode psychosis on present evidence. The framework simply reframes what D2 antagonism is doing, managing the symptomatic consequence of a deeper biology and motivates the search for upstream interventions that could complement or, eventually, partially substitute for it. Second, the framing does not claim that oxidative stress alone causes schizophrenia. Oxidative stress is positioned as an integrative node that connects genetic vulnerability (e.g., C4 complement variants, glutamate-cysteine ligase polymorphisms), developmental disruption, neuroinflammation, mitochondrial dysfunction and neuroplastic compromise. It is the node where multiple upstream pathways converge, and where multiple intervenable mechanisms become available.

## 3 Oxidative stress as an integrative upstream node

The mammalian brain consumes approximately 20% of basal metabolic energy despite representing 2% of body mass, generates reactive oxygen species as a byproduct of mitochondrial oxidative phosphorylation, and depends on an integrated antioxidant network to neutralize them. This network comprises glutathione, superoxide dismutase, catalase, glutathione peroxidases, the thioredoxin and peroxiredoxin systems, and Nrf2-mediated transcriptional regulation of antioxidant gene expression (Halliwell, 2006; Halliwell and Gutteridge, 2015; Hayes and Dinkova-Kostova, 2014). Glutathione is the central low-molecular-weight antioxidant in this network, with dual roles as a direct free-radical scavenger and as the cofactor for glutathione-dependent peroxidases that detoxify hydrogen peroxide and lipid peroxides (Dringen, 2000; Lu, 2013).

Several features make neural tissue unusually vulnerable when this network is impaired. Neurons have high lipid content and large surface area, both of which increase susceptibility to peroxidation. Mitochondrial density and dependence are unusually high in neurons, which means small impairments in mitochondrial function translate quickly into reactive oxygen species accumulation. Neuronal regenerative capacity is limited, so oxidative injury accumulates rather than resolving. Cortical parvalbumin-expressing fast-spiking GABAergic interneurons are particularly susceptible to oxidative injury during development (Cabungcal et al., 2013; Steullet et al., 2017).

The proposition that oxidative stress functions as a convergence point for the genetic and environmental risk factors of schizophrenia is not new, and this framework builds on substantial prior work. Do, Cuenod and colleagues have argued for over a decade that redox dysregulation acts as a central hub, describing feed-forward loops in which redox imbalance, NMDA hypofunction, neuroinflammation and mitochondrial dysfunction amplify one another during development to produce lasting impairment of parvalbumin interneurons and myelinated circuitry (Cuenod et al., 2022; Perkins et al., 2020; Steullet et al., 2017). Palaniyappan et al. (2021) have proposed a glutathione-centered redox-dysregulation subtype. The contribution here is not to restate that oxidative stress is upstream, which that work established, but to integrate the convergence-hub view with the staging architecture of McGorry and the neuroprogression formulation of Berk, to organize the biomarker evidence by stage, and to derive a biomarker-stratified trial design with falsifiable predictions. The trauma-redox-epigenetic proposition in Section 3.4 and the stage-specific intervention logic of Sections 4–6 are where this manuscript extends rather than reiterates the existing redox literature.

The evidence for each component of this node is set out below by domain, with mechanism and measurement treated together rather than separately.

### 3.1 The evidence in brief

The domain-by-domain survey of this evidence is given in full in the accompanying Supplementary Evidence Review, and every study cited there is also tabulated in Supplementary Table S1 with its design, illness stage, treatment status, biomarker source, direction of finding and principal limitation. The pattern across domains can be stated briefly here.

Redox measures are abnormal in ways that connect directly to the proposed mechanism. Brain glutathione is reduced on magnetic resonance spectroscopy (Sydnor and Roalf, 2020; Das et al., 2019), variants in GCLC and GCLM are associated with schizophrenia and with peripheral markers of redox compromise (Gysin et al., 2007; Tosic et al., 2006), and peripheral damage markers are reliably elevated (Ben Othmen et al., 2008; Flatow et al., 2013; Juchnowicz et al., 2021; Mohib et al., 2025), although these peripheral findings, like the inflammatory findings that follow, are open to confounding by smoking and body mass index, which this literature has largely left unmodeled (Section 3.7). Inflammatory markers are elevated, particularly at first episode (Goldsmith et al., 2016; Khandaker et al., 2015), and the kynurenine pathway is dysregulated in a manner coupling inflammation to glutamatergic neurotransmission (Cao et al., 2021; Kindler et al., 2020; Marx et al., 2021; Plitman et al., 2017). Mitochondrial complex I activity is reduced (Ben-Shachar, 2017; Roberts, 2017). Circuit measures show the predicted consequences: mismatch negativity is reduced with the largest effect size among event-related potential candidates (Erickson et al., 2016; Tada et al., 2025), and multivariate imaging classifiers reach approximately 80% sensitivity and specificity (Kambeitz et al., 2015; Di Camillo et al., 2024). Neurotrophic, metabolomic and microbiome findings are reproducible but less specific (Davison et al., 2018; Zheng et al., 2019; Zhu et al., 2020; Yao et al., 2025; Huang et al., 2026).

Three features of this evidence base bear on how it should be read. It is overwhelmingly cross-sectional, a limitation examined in Section 3.7. It compares patients with healthy controls rather than with psychiatric comparison groups, so disorder-specific signatures are largely unestablished. And no single marker is diagnostically specific, and candidate endophenotype markers such as the niacin skin-flush response remain under evaluation (Messamore et al., 2010; Yao et al., 2016); the candidates with the strongest disorder-relative claims are mismatch negativity reduction, elevated striatal presynaptic dopamine synthesis capacity, rare high-penetrance copy-number variants, and reduced prefrontal glutathione in first-episode samples (Erickson et al., 2016; Howes and Kapur, 2009; Marshall et al., 2017). Deployability also differs sharply: high-sensitivity C-reactive protein, IL-6 and peripheral redox markers are standardized and available in routine laboratories today (Chen et al., 2022; Kulaksizoglu and Kulaksizoglu, 2016; Pedraz-Petrozzi et al., 2020); EEG is inexpensive but mismatch negativity and gamma paradigms need protocol standardization; magnetic resonance spectroscopy, positron emission tomography, proteomic and microbiome assays remain research-grade. Two mechanisms central to the framework are shown here rather than in that Supplementary document, because the argument depends on them directly: Figure 3 shows the glutathione-synthesis bottleneck through which genetic variation constrains antioxidant capacity, and Figure 4 shows the kynurenine pathway branch point at which inflammation is converted into altered glutamatergic signaling and further oxidative burden.

**Figure 3:**
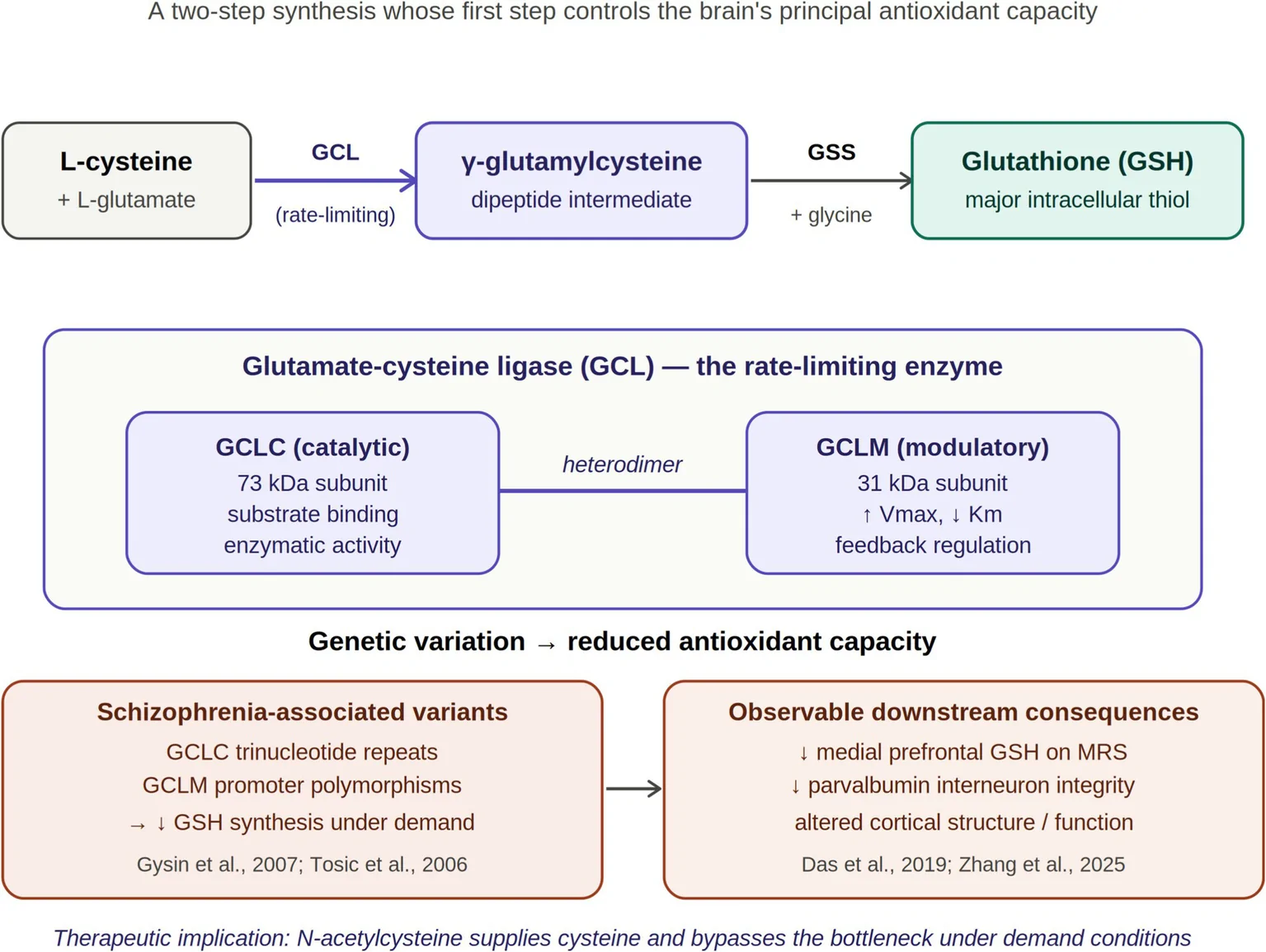
Glutathione synthesis: the GCLC/GCLM rate-limiting bottleneck. Glutathione is synthesized in two steps from L-cysteine and L-glutamate. The first step is catalyzed by glutamate-cysteine ligase (GCL), a heterodimer of catalytic GCLC and modulatory GCLM subunits, and is rate-limiting under demand conditions. Candidate polymorphisms in GCLC and GCLM reported in schizophrenia (Gysin et al., 2007; Tosic et al., 2006) are proposed to reduce capacity, connecting genetic variation to the downstream MRS and cortical phenotype. N-acetylcysteine supplies cysteine and bypasses the bottleneck.

**Figure 4:**
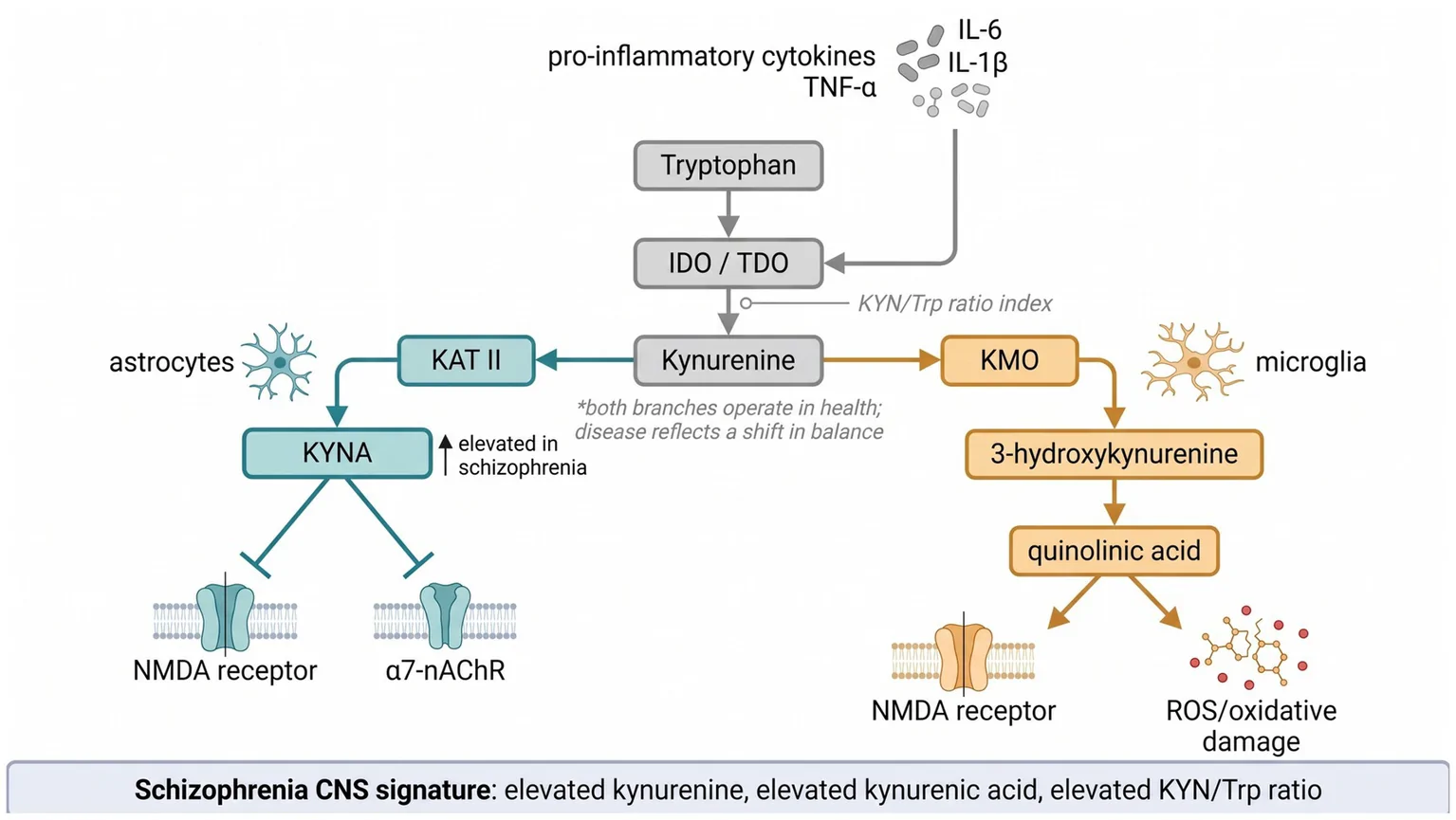
The kynurenine pathway: an inflammation-driven branch point. Pro-inflammatory cytokines induce IDO and TDO, which convert tryptophan to kynurenine. Kynurenine is then metabolized along two competing routes: an astrocytic branch, via KAT II, yielding kynurenic acid, which antagonises NMDA and alpha-7 nicotinic receptors; and a microglial branch, via KMO, yielding 3-hydroxykynurenine and quinolinic acid, which is excitotoxic at NMDA receptors and generates reactive oxygen species. The KYN/Trp ratio indexes pathway activation. Both branches operate in health; in schizophrenia the balance shifts toward the astrocytic branch, and the resulting excess of kynurenic acid contributes to NMDA receptor hypofunction, whereas accumulation of quinolinic acid is more characteristic of neurodegenerative and neuroinflammatory disorders. Abbreviations: IDO, indoleamine 2,3-dioxygenase; TDO, tryptophan 2,3-dioxygenase; KAT II, kynurenine aminotransferase II; KMO, kynurenine 3-monooxygenase; KYNA, kynurenic acid; KYN/Trp, kynurenine to tryptophan ratio; NMDA, N-methyl-D-aspartate; α7-nAChR, alpha-7 nicotinic acetylcholine receptor; ROS, reactive oxygen species; IL-6, interleukin-6; IL-1β, interleukin-1 beta; TNF-α, tumor necrosis factor alpha. Created in BioRender. Lado (2026); https://BioRender.com/jqj8skz

### 3.2 Mitochondria and neurotransmission

Mitochondrial dysfunction is the third arm of the integrative node. Reduced complex I activity, altered mitochondrial DNA copy number and impaired oxidative phosphorylation are reported in post-mortem and peripheral samples (Ben-Shachar, 2017; Roberts, 2017). The connection is bidirectional: mitochondrial inefficiency generates more reactive oxygen species and depletes substrates for glutathione synthesis, while oxidative damage impairs mitochondrial function further. The node also intersects with neurotransmission directly: parvalbumin interneurons express NMDA receptors whose function depends on intact redox biology, and oxidative injury to these cells reduces inhibitory tone on pyramidal neurons, contributing to the gamma-band abnormalities and excitation-inhibition imbalance seen in patients (Cabungcal et al., 2013; Gonzalez-Burgos and Lewis, 2012; Steullet et al., 2017). Figures 5, 6 show the position of complex I in the electron transport chain and the parvalbumin microcircuit, respectively.

**Figure 5:**
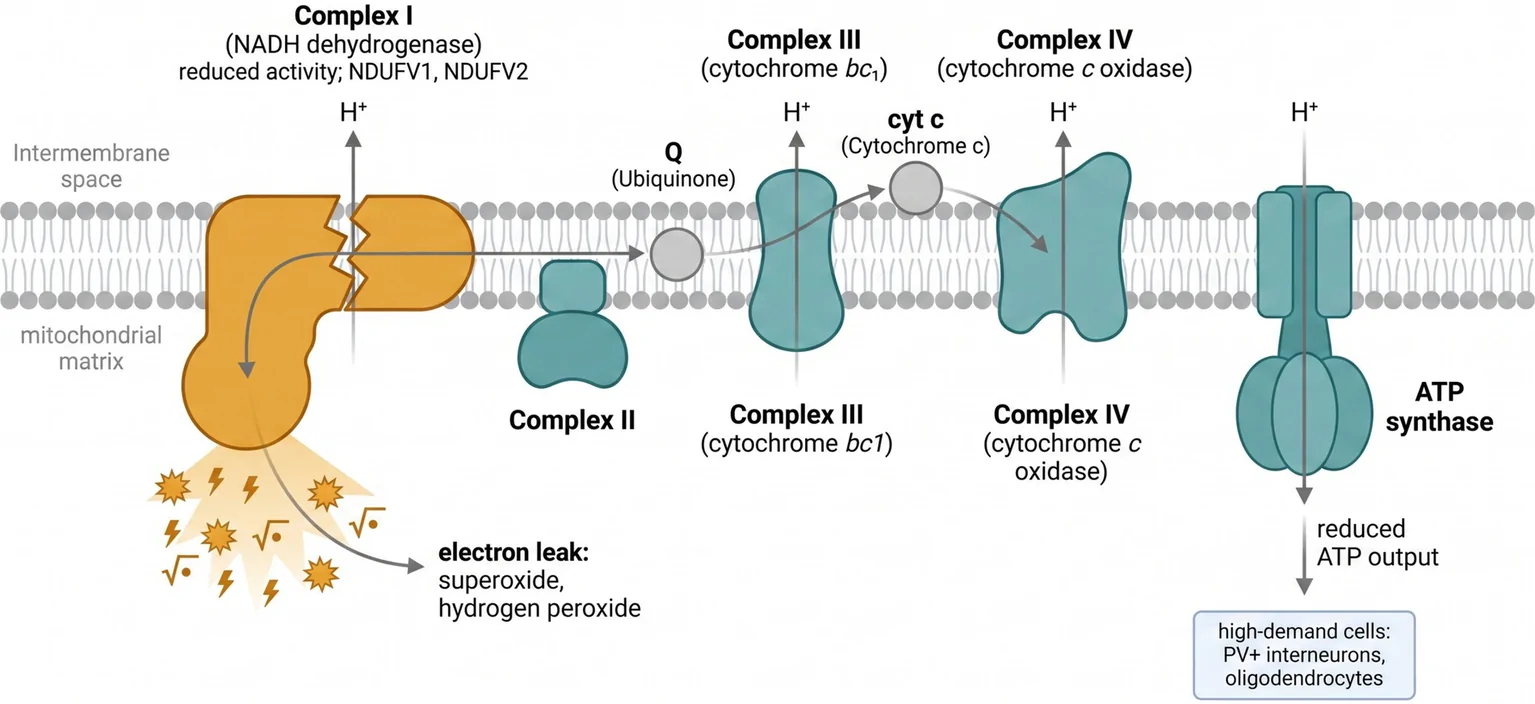
Mitochondrial electron transport chain and complex I deficit in schizophrenia. Complex I (NADH dehydrogenase) is reduced in activity in patients and contributes both to reduced ATP output in high-demand cells (PV + interneurons, oligodendrocytes) and to increased ROS leak (superoxide, hydrogen peroxide) that overwhelms downstream antioxidant defense. Altered expression of NDUFV1, NDUFV2 and other complex I subunits has been documented (Ben-Shachar, 2017). Created in BioRender. Lado (2026); https://BioRender.com/d1mf8r6

**Figure 6:**
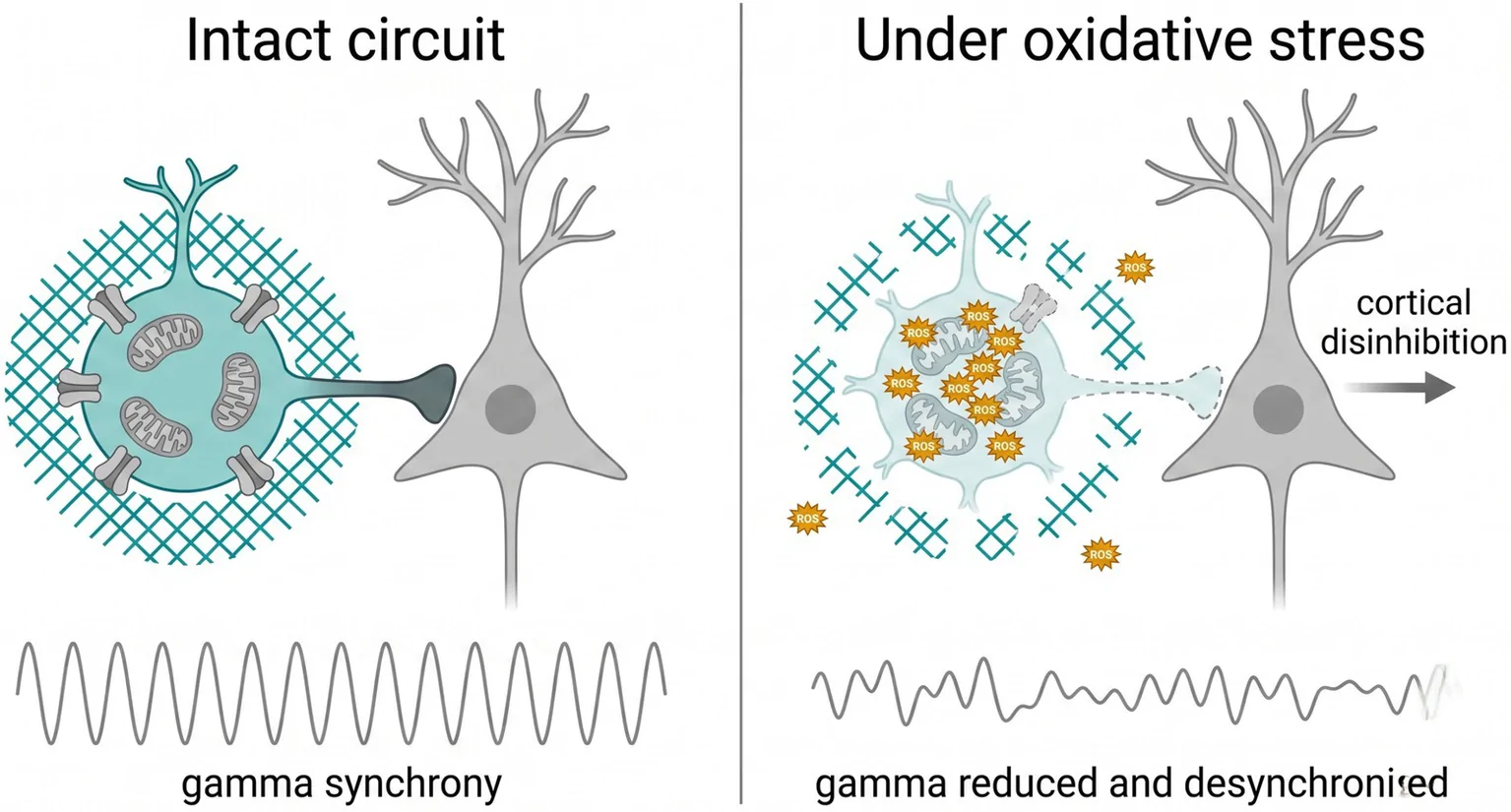
Parvalbumin interneurons: a cellular substrate for upstream redox compromise. Parvalbumin-expressing fast-spiking GABAergic interneurons synchronize pyramidal-cell firing at gamma frequency and are selectively vulnerable to oxidative stress because of their high metabolic demand, limited intrinsic antioxidant buffering, and redox-sensitive perineuronal nets. Under oxidative load, PV-interneuron excitability falls, perineuronal nets degrade, gamma synchrony reduces and desynchronizes, NMDA tone drops on PV cells, and dopaminergic disinhibition follows (Cabungcal et al., 2013, 2014; Steullet et al., 2017; Gonzalez-Burgos and Lewis, 2012; Coyle, 2012). Created in BioRender. Lado (2026); https://BioRender.com/b8fgsu6

Brain-derived neurotrophic factor (BDNF), the principal neurotrophins supporting synaptic plasticity and neurogenesis, is the fourth element. Peripheral BDNF is moderately reduced in schizophrenia compared with healthy controls, with effect sizes in the range of −0.5 to −0.7 (Ahmed et al., 2021; Dombi et al., 2022; Fernandes et al., 2015; Green et al., 2011). The reduction is observed in medicated and drug-naïve patients alike. Oxidative stress reduces BDNF expression and signaling; the inflammatory state reduces it further; and reduced BDNF compromises the neuroplastic capacity that would otherwise support recovery from these insults. The mechanistic case for the redox-inflammation-neurotrophins loop as a single integrated upstream node, rather than as three parallel mechanisms, is increasingly well supported.

### 3.3 Circuit-level readouts

Two classes of measurement index the circuit-level consequences of the biology described above: structural and neurochemical imaging, and electrophysiology.

#### 3.3.1 Imaging

Univariate structural MRI findings (reduced gray matter in the superior temporal gyrus, hippocampus and anterior cingulate, and enlarged ventricles) are highly replicated but lack diagnostic specificity at the individual level. Multivariate pattern recognition has improved diagnostic performance substantially. A meta-analysis of 38 studies including 1,602 patients and 1,637 controls reported pooled sensitivity and specificity of approximately 80% (Kambeitz et al., 2015); a more recent meta-analysis across structural MRI, resting-state fMRI, task fMRI and diffusion tensor imaging reproduced this approximate 80% ceiling and identified disease stage, antipsychotic exposure and methodological choices as the principal moderators (Di Camillo et al., 2024). In CHR samples, multimodal MRI signatures distinguish individuals who transition from those who do not at accuracies in the 70–85% range, exceeding any clinical predictor alone (Di Camillo et al., 2024).

Proton magnetic resonance spectroscopy provides *in vivo* neurochemistry: the largest synthesis to date (92 studies, 2,721 controls, 2,822 patients) reported elevated glutamate in basal ganglia, frontal cortex and medial prefrontal cortex (Lopes et al., 2024), and medial prefrontal glutathione is reduced, with a pooled effect of d = −0.21 in ultra-high-field studies (Sydnor and Roalf, 2020) against the 52% reduction in the original *in vivo* report (Do et al., 2000). GABA reductions in midcingulate cortex have been reported in first-episode psychosis (McCutcheon et al., 2020). Figure 7 summarizes the regions with reproducible findings. An emerging and uniquely accessible target is the retina, the only central nervous system tissue that can be imaged directly and non-invasively in living patients: meta-analysis across 65 studies documents consistent thinning of the peripapillary retinal nerve fiber layer and macular ganglion-cell layers, with thinning associated with illness duration and symptom severity (Komatsu et al., 2024). Retinal measures remain non-specific and are confounded by diabetes and hypertension, so their value for stage tracking depends on establishing specificity.

**Figure 7:**
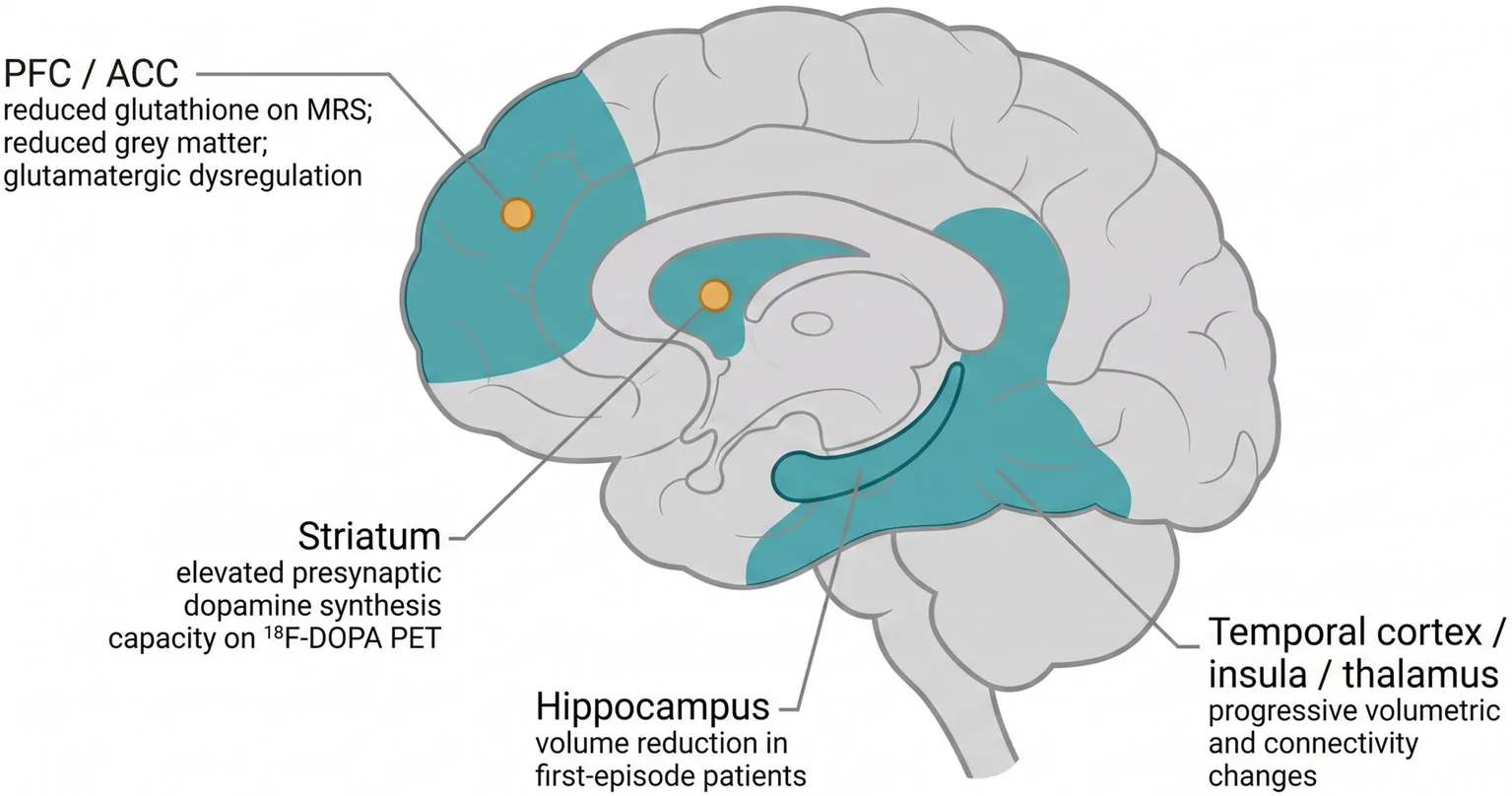
Brain regions with reproducible findings in schizophrenia. Stylized sagittal schematic showing PFC/ACC (reduced GSH on MRS, reduced gray matter, glutamatergic dysregulation), hippocampus (volume reduction in first-episode patients), striatum (elevated presynaptic dopamine synthesis capacity on ^18^F-DOPA PET), and temporal cortex/insula/thalamus (progressive volumetric and connectivity changes). Schematic only; not derived from imaging data (Das et al., 2019; Lopes et al., 2024; Guo et al., 2014; Howes and Kapur, 2009; McCutcheon et al., 2019). Created in BioRender. Lado (2026); https://BioRender.com/7xsbz08

The regional findings should be read against the cellular claim of Section 3.1 rather than as a list of separate sites of injury. Parvalbumin interneurons are distributed throughout the cortex, so the framework predicts graded rather than focal involvement, weighted toward regions where metabolic demand is high and antioxidant reserve is low. The anterior cingulate is where the lines of evidence converge most directly. It is the region in which pooled magnetic resonance spectroscopy studies show reduced glutathione in schizophrenia, a small deficit that runs in the opposite direction in bipolar disorder (Das et al., 2019), and the region in which oxidative stress tracked the loss of parvalbumin interneuron integrity across genetic and environmental animal models (Steullet et al., 2017). Postmortem abnormalities of parvalbumin neurons in schizophrenia are likewise cortical, although which cell types carry the underlying signaling deficit remains unresolved (Gonzalez-Burgos and Lewis, 2012). The striatal finding has a different status: elevated presynaptic dopamine synthesis is better read as the downstream expression of the dopaminergic disinhibition shown in Figure 6 than as a second site of oxidative injury. The hippocampal and temporal findings sit between these; the same interneuron population operates there, but the evidence linking hippocampal change to redox status in patients is limited to the interaction reported by Alameda et al. (2018). None of this can be localized from peripheral markers, which is a further reason that the markers discussed in Section 3.6 index systemic rather than regional disturbance.

#### 3.3.2 Electrophysiology

Mismatch negativity (MMN), an automatic auditory event-related potential evoked by deviant stimuli is among the most replicated electrophysiological findings in schizophrenia, with the largest effect size among ERP candidates (Erickson et al., 2016; Tada et al., 2025). MMN is generated by NMDA-receptor-dependent mechanisms and has been replicated in animal models, making it a translational biomarker that connects directly to the upstream NMDA-hypofunction component of the framework. Notably, MMN amplitude deficits are associated with future transition to psychosis among CHR individuals, although the associations are modest and MMN shows limited predictive value on its own (Hamilton et al., 2022). The disorder-relative specificity is also relatively favorable: MMN deficits are smaller in bipolar.

disorder than in schizophrenia (Erickson et al., 2016; Tada et al., 2025). A systematic review of EEG-based measures in at-risk and early schizophrenia identified reduced MMN and P300 amplitudes and impaired sensory gating as the most consistent abnormalities (Perrottelli et al., 2021). Figure 8 shows both readouts: the reduced mismatch negativity in panel A and the attenuated, phase-jittered gamma entrainment in panel B, each linking the electrophysiological signal back to the parvalbumin interneuron substrate shown in Figure 6.

**Figure 8:**
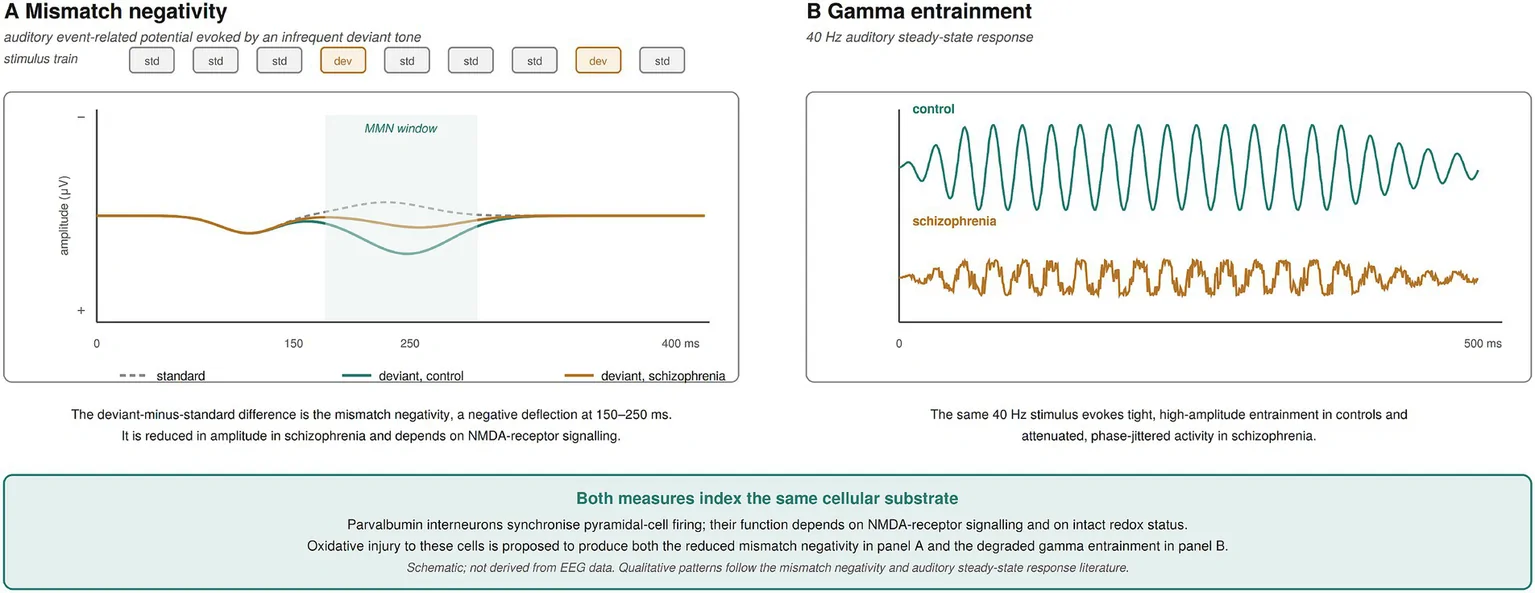
Auditory electrophysiology: two circuit-level readouts of parvalbumin interneuron integrity. **(A)** Mismatch negativity. In an oddball sequence, an infrequent deviant tone evokes a negative deflection at 150–250 ms relative to the standard tone. This deviant-minus-standard difference is the mismatch negativity; it depends on NMDA-receptor signaling and is reduced in amplitude in schizophrenia, with the largest effect size among event-related potential candidates (Erickson et al., 2016; Tada et al., 2025). **(B)** Gamma entrainment. A 40 Hz auditory stimulus evokes tight, large-amplitude entrainment in controls, generated by parvalbumin-interneuron-driven synchronization of pyramidal cells (Gonzalez-Burgos and Lewis, 2012); the same stimulus produces attenuated, phase-jittered activity in schizophrenia. Both measures index the same cellular substrate, whose function depends on NMDA-receptor signaling and intact redox status. Schematic; not derived from EEG data. The qualitative patterns follow the mismatch negativity and auditory steady-state response literature.

### 3.4 The genetic substrate

Schizophrenia is highly heritable, around 80 percent, but for this framework the value of its genetics lies less in explaining causation than in enabling detection: the variants that make a person vulnerable to the upstream cascade can in principle be identified before symptoms appear. The architecture is diffuse, with hundreds of common variants of small effect alongside rarer mutations of larger effect (Trubetskoy et al., 2022; Marshall et al., 2017), but a subset is directly informative here. A variant in the complement C4 gene affects synaptic pruning during development, tying inherited risk to developmental disruption (Sekar et al., 2016). More directly, common variants in the glutathione-synthesis genes GCLC and GCLM have been associated with schizophrenia and with peripheral markers of redox compromise (Gysin et al., 2007; Tosic et al., 2006). These are candidate-gene findings from small cohorts rather than genome-wide significant loci, and they should be read as mechanistically informative modifiers of redox capacity, not as validated genetic risk markers. Their interest lies in what they propose about mechanism: if a low-capacity genotype imposes a reduced ceiling on antioxidant production, it would predispose to precisely the imbalance this framework places upstream. That proposition is testable (Section 7.3 states the form such a test would take), but it is not yet a basis for clinical screening. The point is not that any single gene causes the illness, but that genetic vulnerability marks one plausible route by which the redox node becomes compromised.

Figure 9 shows both routes and their convergence, together with the interaction between inherited antioxidant capacity and environmental insult.

**Figure 9:**
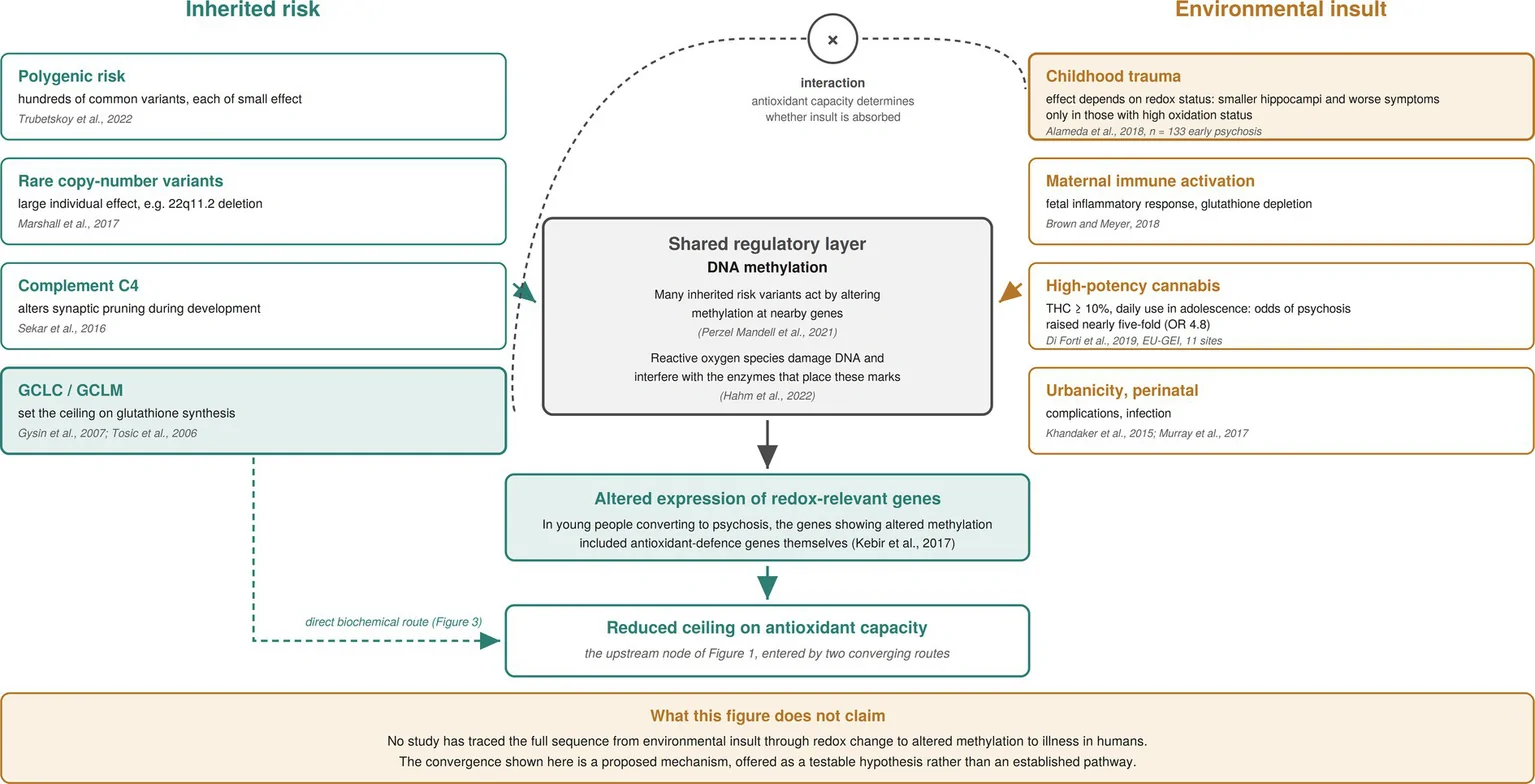
The genetic substrate and its convergence with environmental insult. Inherited risk reaches the redox node by two routes. The direct biochemical route runs through GCLC and GCLM, which encode the subunits of glutamate-cysteine ligase and set the ceiling on glutathione synthesis (Gysin et al., 2007; Tosic et al., 2006; see Figure 3). The indirect route runs through DNA methylation: many inherited risk variants appear to act by altering methylation at nearby genes (Perzel Mandell et al., 2021), and reactive oxygen species damage DNA in ways that interfere with the enzymes placing and removing these marks (Hahm et al., 2022). Environmental insults converge on the same regulatory layer, and in young people converting to psychosis the genes showing altered methylation included antioxidant-defense genes themselves (Kebir et al., 2017). The arc across the top marks the interaction rather than a separate pathway: in 133 patients with early psychosis, childhood trauma predicted smaller hippocampal volume and more severe symptoms only in those with high oxidation status (Alameda et al., 2018), indicating that inherited antioxidant capacity determines whether an environmental insult is absorbed or converted into lasting change. No study has traced the full sequence from environmental insult through redox change to altered methylation to illness in humans; the convergence shown is a proposed mechanism offered as a testable hypothesis.

Genes are not the whole story. The same substrate is shaped by epigenetics: chemical marks, chiefly DNA methylation, that change how strongly a gene is expressed without altering the sequence. What matters here is that oxidative stress can disturb this system directly: reactive oxygen species damage DNA in ways that interfere with the enzymes placing and removing these marks, so rising oxidative stress can re-set which genes are active (Hahm et al., 2022). This is not merely theoretical. In young people transitioning to psychosis, the genes showing altered methylation included antioxidant-defense genes themselves, tying the epigenetic change back to redox biology (Kebir et al., 2017). Childhood trauma illustrates the convergence: it interacts with redox status to predict brain and clinical outcomes in early psychosis (Alameda et al., 2018), consistent with an environmental insult activating a latent, genetically-set vulnerability rather than acting on a blank slate. Many inherited risk variants also appear to act by changing methylation at nearby genes, so inherited risk and environmental insult converge on one regulatory layer (Perzel Mandell et al., 2021). The evidence does not show that oxidative stress switches on latent schizophrenia genes, but it does show that redox status shapes their expression, and this is one concrete mechanism through which genes and environment interact.

### 3.5 Developmental and environmental origins

Infection is a further environmental input converging on the same upstream biology, and the epidemiological evidence is considerably stronger than the case literature that first drew attention to the link. Childhood central nervous system viral infection is associated with an approximately two-fold increased risk of adult non-affective psychosis (risk ratio 1.70), while bacterial CNS infection is not (Khandaker et al., 2012); maternal infection during gestation raises offspring risk of non-affective psychosis (relative risk 1.28) and of schizophrenia specifically (relative risk 1.65), with second-trimester exposure carrying the greatest risk (Saatci et al., 2021). The mechanism implicated is not usually direct infection of the developing brain but maternal immune activation: elevated maternal cytokines and the resulting fetal inflammatory response are proposed to drive microglial activation, oxidative stress and glutathione depletion, converging on the parvalbumin-interneuron and redox endpoints described above, with animal models reproducing the dopaminergic and NMDA-related phenotypes (Brown and Meyer, 2018). Infection is therefore an additional environmental entry point into the same cascade rather than a separate causal pathway, and helps explain why inflammatory and redox markers are elevated well before psychotic symptoms emerge. Figure 10 summarizes this convergence.

**Figure 10:**
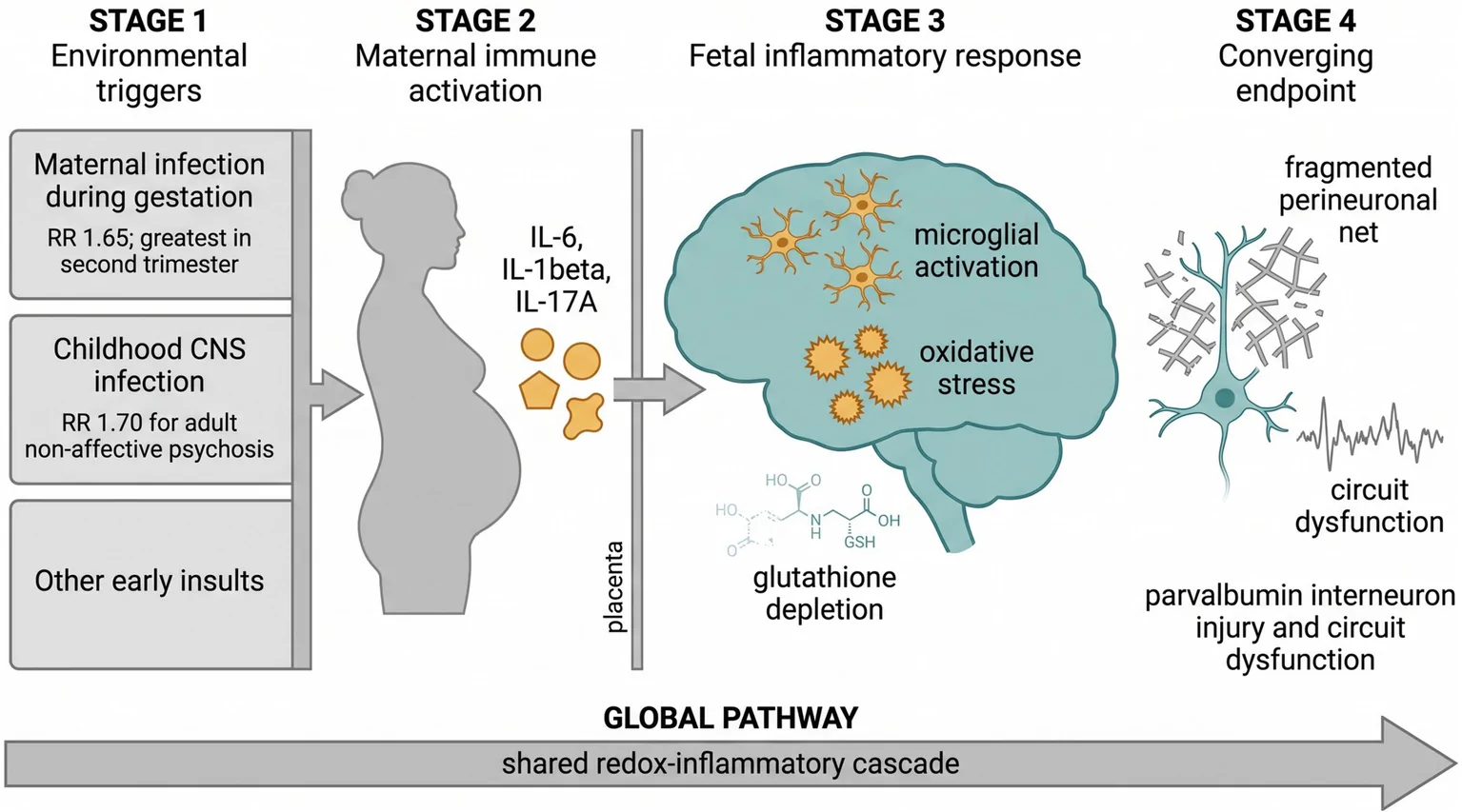
Maternal immune activation as an upstream entry point. Environmental triggers, including maternal infection during gestation, childhood central nervous system infection, and other early insults, converge via maternal immune activation on the shared redox-inflammatory cascade placed upstream in this framework. Elevated maternal cytokines (IL-6, IL-1β, and IL-17A) and the resulting fetal inflammatory response drive microglial activation, oxidative stress and glutathione depletion in the developing brain, converging on the same parvalbumin-interneuron injury and circuit dysfunction described elsewhere. Risk ratios shown are drawn from the meta-analyses cited in the text: childhood CNS viral infection and adult non-affective psychosis (risk ratio 1.70; Khandaker et al., 2012); maternal infection and offspring schizophrenia (relative risk 1.65, greatest with second-trimester exposure; Saatci et al., 2021); mechanistic and animal-model evidence for maternal immune activation (Brown and Meyer, 2018). Schematic; not derived from primary data. Created in BioRender. Lado (2026); https://BioRender.com/fql4cu9

Cannabis illustrates how sharply the environmental contribution depends on exposure characteristics rather than exposure alone. In the EU-GEI multicenter case–control study, daily use of high-potency cannabis, defined as a tetrahydrocannabinol content of 10% or more, carried nearly five-fold increased odds of psychotic disorder (adjusted odds ratio 4.8) against 3.2 for daily use of any cannabis; assuming causality, the population attributable fraction reached 30% in London and 50% in Amsterdam (Di Forti et al., 2019), consistent with an earlier south London series in which daily use of high-potency preparations carried an adjusted odds ratio of 5.4 (Di Forti et al., 2015). Potency and frequency, not cannabis exposure as such, carry the association, and the adolescent window in which use typically begins coincides with the period of parvalbumin interneuron maturation described above.

### 3.6 Synthesis and specificity

Oxidative stress, neuroinflammation, mitochondrial dysfunction and reduced neurotrophic signaling are not unique to schizophrenia; the same processes are implicated in bipolar disorder, major depression and several neurodegenerative diseases. Is this a theory of schizophrenia, or of chronic brain illness in general? Our position is that the upstream machinery is largely shared, and that disorder-specificity arises not from the existence of redox-inflammatory compromise but from where, when and in which cell populations it acts, together with the genetic and developmental context. In schizophrenia specifically, the convergence on parvalbumin interneurons and gamma-generating microcircuitry during adolescence, the association with GCLC/GCLM genetics and the C4-linked pruning pathway, and the more disorder-associated findings (mismatch negativity reduction, elevated striatal presynaptic dopamine and CSF kynurenic acid) are what distinguish it. The framework is a disorder-specific instantiation of a partly transdiagnostic upstream biology. This also means the specificity of any single peripheral marker will remain limited, and differentiation from bipolar and psychotic depression must be established empirically rather than assumed.

No individual biomarker meets stringent diagnostic specificity criteria. The candidates with the strongest disorder-relative specificity claims are MMN reduction; elevated striatal presynaptic dopamine synthesis capacity on [18F]-DOPA PET; rare high-penetrance CNVs when present; and reduced prefrontal glutathione on MRS in first-episode samples (Erickson et al., 2016; Howes and Kapur, 2009; Marshall et al., 2017; Zhang et al., 2025). The realistic short-term translational goal is not a single schizophrenia test but a multimodal panel combining imaging, electrophysiology, peripheral inflammation and redox markers, BDNF and polygenic risk score (Lozupone et al., 2017; Zaki et al., 2022).

Peripheral and central redox measures are not interchangeable, and the literature moves between them more freely than the evidence allows. Erythrocyte glutathione, malondialdehyde and advanced oxidation protein products index systemic redox status; magnetic resonance spectroscopy indexes cortical glutathione. Where the two have been measured in the same people, the relationship has not been stable. In one early-psychosis sample, medial prefrontal glutathione correlated positively with blood glutathione peroxidase activity in male controls but negatively in male patients (Xin et al., 2016), and medial prefrontal glutathione was not reduced in an antipsychotic-naive clinical high-risk sample in which plasma glutathione peroxidase was also measured (Da Silva et al., 2018). Peripheral markers are therefore treated here as indices of systemic disturbance that may accompany central compromise, not as proxies for it, and they carry less weight than central measures wherever the argument concerns brain biology. Damage products such as malondialdehyde also index oxidative damage, which is a separate quantity from the current state of redox regulation (Lado, 2026), a further reason not to read them as measures of the regulatory disturbance this framework proposes.

This lack of specificity matters most at Risk Strata 0 and 1 and at Stage 2, where symptoms overlap heavily with anxiety and depressive disorders, and where this framework proposes intervening. No single marker should therefore carry diagnostic weight, and the case for a panel rests on combining markers that carry partly independent information.

That case has to be stated with its assumption visible, because the assumption is in tension with the framework itself. Take a help-seeking group in which one person in four develops psychosis within 2 years, roughly what the transition literature reports, and suppose for illustration that each abnormal marker multiplies the odds of transition by about 2.3 and each normal marker by about 0.59. If three markers were conditionally independent given outcome, one abnormal marker would raise estimated risk to just under one in two, three abnormal markers to about four in five, and three normal markers would lower it to about one in sixteen. Conditional independence is what licenses multiplying the three. But this framework proposes that redox, inflammatory and circuit disturbance form one self-reinforcing loop with a common upstream node, in which case the markers are correlated and multiplication overstates what they convey together. The two positions cannot both be held, and the independence figures should be read as an upper bound.

A sensitivity analysis makes the dependence explicit. Treating correlated markers as equivalent to a smaller number of independent ones, three markers carrying the information of two independent markers give an estimated risk of about 64% when all are abnormal and about 10% when all are normal; three markers carrying the information of one give about 43 and 16%. The realistic range lies between those bounds, and where a given panel falls within it is an empirical question that requires estimating the correlation between markers in the same individuals. The per-marker figures are themselves illustrative. For medial prefrontal glutathione, the most direct available measure of the proposed mechanism, the pooled effect of d = −0.21 (Sydnor and Roalf, 2020) corresponds to an area under the curve of about 0.56 and, at a symmetric cutoff, to an odds multiplier of about 1.2 for an abnormal result; a multiplier of 2.3 would require an effect near d = 1.0. Glutathione therefore cannot carry a classification on its own. The derivations for this paragraph and the preceding one are given in the Supplementary Statistical Note. What this means in practice is that no single redox measure now available should change what a clinician does with an individual patient. At an area under the curve of 0.56, an abnormal glutathione value moves a baseline risk of one in four to just under three in ten, which is not enough to begin an intervention, and a normal value is not enough to stop monitoring.

Ruling out remains the more defensible near-term use, because the two errors are not equivalent. Even at the most optimistic bound, a positive panel misidentifies one person in five, and that error costs a young person years of surveillance for an illness that never arrives. A negative panel may reasonably support stepping monitoring down, but under correlated markers the residual risk after a negative panel is closer to one in ten than to one in sixteen.

Deployability differs sharply across these domains: high-sensitivity C-reactive protein, IL-6 and peripheral redox markers are standardized and available in routine laboratories today; EEG is inexpensive but mismatch negativity and gamma paradigms need protocol standardization; and magnetic resonance spectroscopy, positron emission tomography, proteomic and microbiome assays remain research-grade; neuromelanin-sensitive MRI additionally requires cross-scanner harmonization before multisite use (Wengler et al., 2021).

### 3.7 The causal claim and its limits

This framework makes two claims that should be separated. The first is that redox compromise acts early in the process and can be modified there. The second, stronger claim would be that redox compromise causes schizophrenia. Only the first is made here, and the distinction matters, because the human evidence bears on the two unequally. Nearly all of it is cross-sectional: patients measured once against healthy controls, differing in illness duration and medication exposure, both of which affect the measures in question. Such designs cannot establish which came first, cannot show that the abnormality produced the clinical change, and cannot exclude that it follows from the illness or its treatment. Nor is the abnormality specific: raised damage markers and reduced antioxidant capacity are reported in mood disorders, anxiety disorders and neurodegenerative disease.

Health behaviors add a layer of confounding that this literature has largely left unmodeled, and smoking is the most important. People with schizophrenia are several times more likely to smoke than the general population, with a weighted odds ratio for current smoking of 5.9 across 42 studies in 20 countries, and the authors of that synthesis concluded that people who go on to develop schizophrenia carry risk factors that make them more likely to start smoking (de Leon and Diaz, 2005). Tobacco smoke is an oxidant load, yet the direction of its effect on peripheral markers in this population is not predictable: in one sample of male inpatients with schizophrenia, malondialdehyde was lower in smokers than in nonsmokers (Zhang et al., 2007). A confounder of unknown direction cannot be corrected by assumption, so every peripheral redox finding cited here should be read as potentially affected by smoking, and body mass index, diet and physical activity carry the same caveat. All four are specified as covariates in the trial design in Section 6.4.

A distinction is worth drawing, however, between differences that confound the comparison and differences that may belong to the same process. Metabolic disturbance is the clearest case. In a meta-analysis of twelve studies and 1,137 participants, antipsychotic-naive patients with first-episode psychosis showed elevated insulin resistance and a 5.4-fold increase in the odds of impaired glucose tolerance compared with matched controls (Perry et al., 2016). Because these patients had never received antipsychotic medication, the finding cannot be attributed to treatment, and because it is present at first episode it cannot be attributed to years of illness or inactivity. Metabolic variability is also greater in this group than in controls, consistent with a subgroup in whom the disturbance is marked (Pillinger et al., 2022).

The implication is direct. If a systemic redox-inflammatory disturbance is present, then metabolic dysfunction and psychosis may be parallel expressions of it rather than one causing the other, and treating metabolic status purely as a nuisance variable would discard information. The authors of the glycaemic meta-analysis framed their question in these terms, asking whether schizophrenia and type 2 diabetes share intrinsic disease pathways that cause them to develop in unison. The framework advanced here is compatible with that reading. Medication exposure and illness duration remain genuine confounders. Metabolic status may not be one.

Evidence about direction is limited but not absent. In rodents, depleting glutathione experimentally damages parvalbumin interneurons and disrupts gamma activity. Exposing neuronal cells to oxidative challenge disrupts mitochondrial quality control in the predicted direction, alongside matching abnormalities in patients’ white blood cells (Li et al., 2026). In humans, one prospective study measured erythrocyte glutathione in 36 at-risk individuals and followed them for 7 years: low baseline glutathione was significantly associated with transition to psychosis, with an area under the curve of 0.819 (Lavoie et al., 2017). This is the design that establishes temporal precedence, and its direction supports the framework. It is a single small sample, unreplicated, using a peripheral rather than a central measure, and it cannot carry the causal claim on its own. What it does establish is that the question is answerable, and that the one time it was asked this way, the answer pointed the direction this framework predicts.

The animal evidence also runs the other way, and the strongest case against the ordering proposed here comes from the same evidence tier on which that ordering rests. Repeated exposure to the NMDA receptor antagonist ketamine produces a persistent rise in brain superoxide through neuronal NADPH oxidase, with loss of the parvalbumin and GAD67 phenotype in fast-spiking prefrontal interneurons, and reducing superoxide production prevents that loss (Behrens et al., 2007). In that model, oxidative stress lies downstream of NMDA receptor hypofunction, not upstream of it. Two things follow. The directional claim cannot be sustained on animal evidence, which supports both orderings. And the same experiment supports the claim this framework actually makes, since intervening at the redox step prevented the interneuron damage regardless of what initiated it.

The framework therefore does not depend on a one-way causal chain. Rather than oxidative stress causing inflammation, which causes NMDA hypofunction, which causes circuit failure, these processes form a loop that feeds itself: oxidative burden, inflammation, inefficient mitochondria and disturbed excitatory-inhibitory balance each make the others worse, and damage anywhere increases strain everywhere. The claim is not that redox compromise is the first or only cause, but that it is an early point of entry into the loop, that it can be measured, and that it can be modified more easily there than further downstream. This is a weaker claim, and it is the one the evidence supports. It also means the framework does not rest on the position of any single node. NMDA hypofunction is one node among several, alongside GABAergic, excitatory-inhibitory and neuroimmune disturbance, which may run in parallel with it rather than follow from it.

One study would move this from hypothesis toward evidence: replication of that prospective result, low baseline glutathione predicting later transition, in a larger multi-site cohort measuring glutathione centrally as well as peripherally (Lavoie et al., 2017). A cohort of that design answers two questions at once, whether the finding holds and, through mediation analysis within it, whether redox change accounts for the clinical change that follows. No cohort of that size currently exists. The therapeutic claim is tested separately, by the trial design in Section 6.

A word on what this model is for. Oxidative stress is not specific to schizophrenia. It appears in mood disorders, in neurodegenerative disease, and in chronic illness generally, and that ubiquity is usually treated as a reason to discount it. It may be the more interesting observation, because it raises a question the field has not asked: what determines which illness follows, and in what order. Schizophrenia has been studied exhaustively as a set of symptoms, as a diagnosis, and as biological correlates measured at single timepoints. It has rarely been studied as a process unfolding in time, with redox biology treated as something that might drive that process rather than accompany it. This framework exists to make that question answerable.

### 3.8 The case against progression

The progressive model of schizophrenia is contested, and the case against it is strong enough that the framework has to meet it directly. A critical review of longitudinal clinical, imaging and cognitive studies concluded that although roughly a quarter of people with schizophrenia have a poor long-term outcome, few show the incremental loss of function characteristic of neurodegenerative disease; that the further decreases in brain tissue volume after onset are explicable by antipsychotic medication, substance use and other secondary factors; and that cognitive functioning, though impaired relative to controls, does not appear to deteriorate over time (Zipursky et al., 2013). The imaging evidence on medication is specific. In 211 patients followed with repeated scans over an average of 7.2 years, greater intensity of antipsychotic treatment was associated with smaller gray matter volumes after controlling for illness duration, severity and substance misuse (Ho et al., 2011). Treatment intensity in observational cohorts is chosen partly in response to illness, so residual confounding by indication cannot be excluded, and the meta-analysis that follows shares that limitation; the primate evidence does not. A meta-analysis of 30 longitudinal studies found progressive gray matter decreases related to cumulative antipsychotic exposure but not to illness duration or severity (Fusar-Poli et al., 2013b). In macaques given haloperidol or olanzapine for 17 to 27 months at clinically comparable plasma levels, brain weight and volume fell by 8 to 11% in the absence of any illness (Dorph-Petersen et al., 2005).

This evidence narrows what the word progressive can mean here. It cannot mean inevitable, lifelong neurodegeneration, and the framework does not claim that. Cognitive impairment is largely established by the first years of illness (Section 7.4), and the late decline reported in severe treatment-resistant illness (Pathak et al., 2026) concerns a subgroup consistent with the quarter who fare poorly, not the course of the illness as a whole. What is proposed is a process concentrated in the years before and around onset, in which movement from risk to psychosis is accompanied by measurable change in redox and circuit biology, and in which intervention could alter what follows. Structural change after onset is not used as evidence for that process, because in treated cohorts medication cannot be separated from illness.

The progressive framing would be abandoned under any of three results: if upstream redox and circuit markers, measured repeatedly within individuals in antipsychotic-naive at-risk cohorts, show no change across transition; if structural change after onset proves fully accounted for by medication and secondary factors in cohorts with adequate control for them; or if the rate of change in those markers does not distinguish people who transition from those who do not. Under any of these, progression is the wrong description of the illness, and the staging structure should be retained only as a classification of risk, without a mechanistic claim attached.

## 4 A five-stage classification with intervention windows

The framework set out above describes a process that unfolds over time. If that description is correct, it should be possible to say where along that process a given patient sits, what biology is most informative at that point, and what intervention is proportionate to it. This section sets out a five-level classification that attempts exactly that, using McGorry’s stage numbering with the first two levels renamed as risk strata to avoid implying early disease. Figure 11 shows the trajectory the framework proposes, the intervention windows aligned to it, and the biomarkers most appropriate to measure at each level; the sections that follow define each level and the evidence bearing on it.

**Figure 11:**
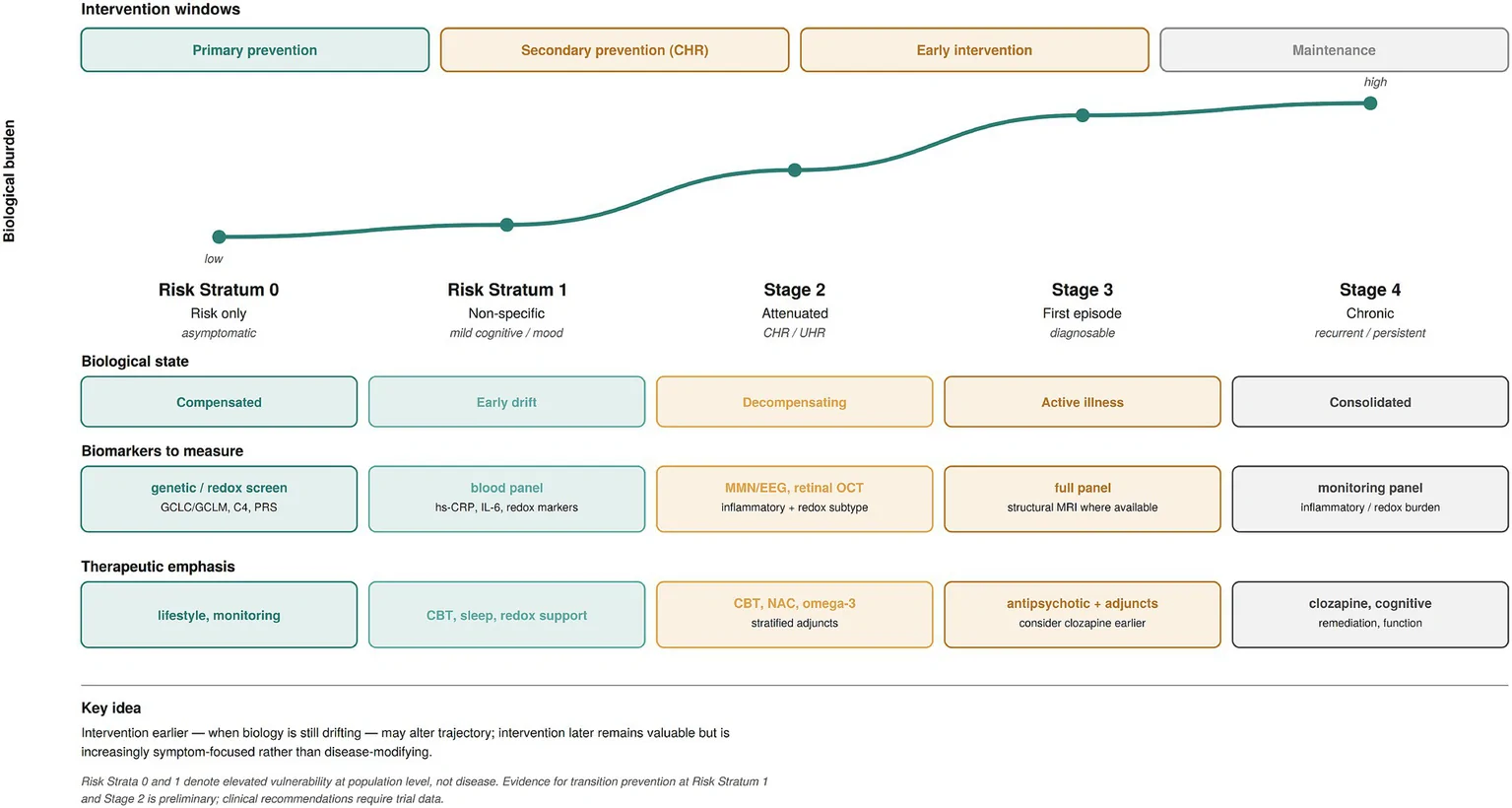
Disease trajectory and intervention windows across the two risk strata and three stages, with the biomarkers most appropriate to measure at each stage. The biomarker row shows a practical, stage-proportionate panel; the specific tests are detailed in this section. Abbreviations: GCLC/GCLM, catalytic and modifier subunits of glutamate-cysteine ligase; C4, complement component 4; PRS, polygenic risk score; hs-CRP, high-sensitivity C-reactive protein; IL-6, interleukin-6; MMN, mismatch negativity; EEG, electroencephalography; OCT, optical coherence tomography; MRI, magnetic resonance imaging; NAC, N-acetylcysteine; CHR/UHR, clinical/ultra-high risk.

The classification proposed here uses McGorry’s five-stage numbering and adds explicit anchoring to the biomarker domains summarized in Section 3. Figure 12 summarizes the clinical features defining each level, and Figure 13 illustrates the biomarker subtypes that may refine classification within stage.

**Figure 12:**
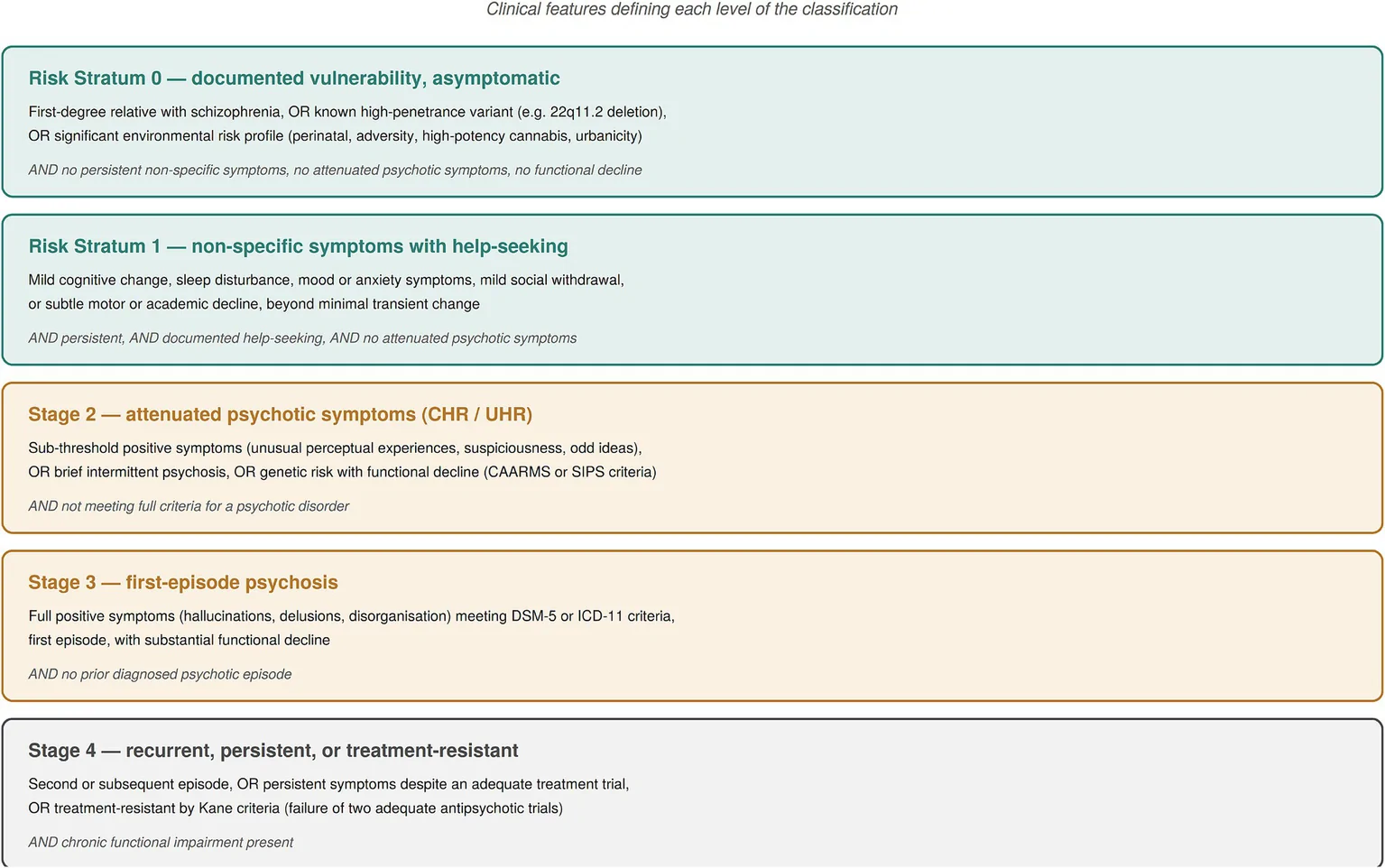
Clinical features defining each level. Entry to Risk Stratum 1 requires persistent non-specific symptoms, beyond the minimal transient change common in adolescence, together with documented help-seeking; a single subjectively reported symptom is not sufficient. Operational thresholds are deliberately not specified here and will be set and validated separately.

**Figure 13:**
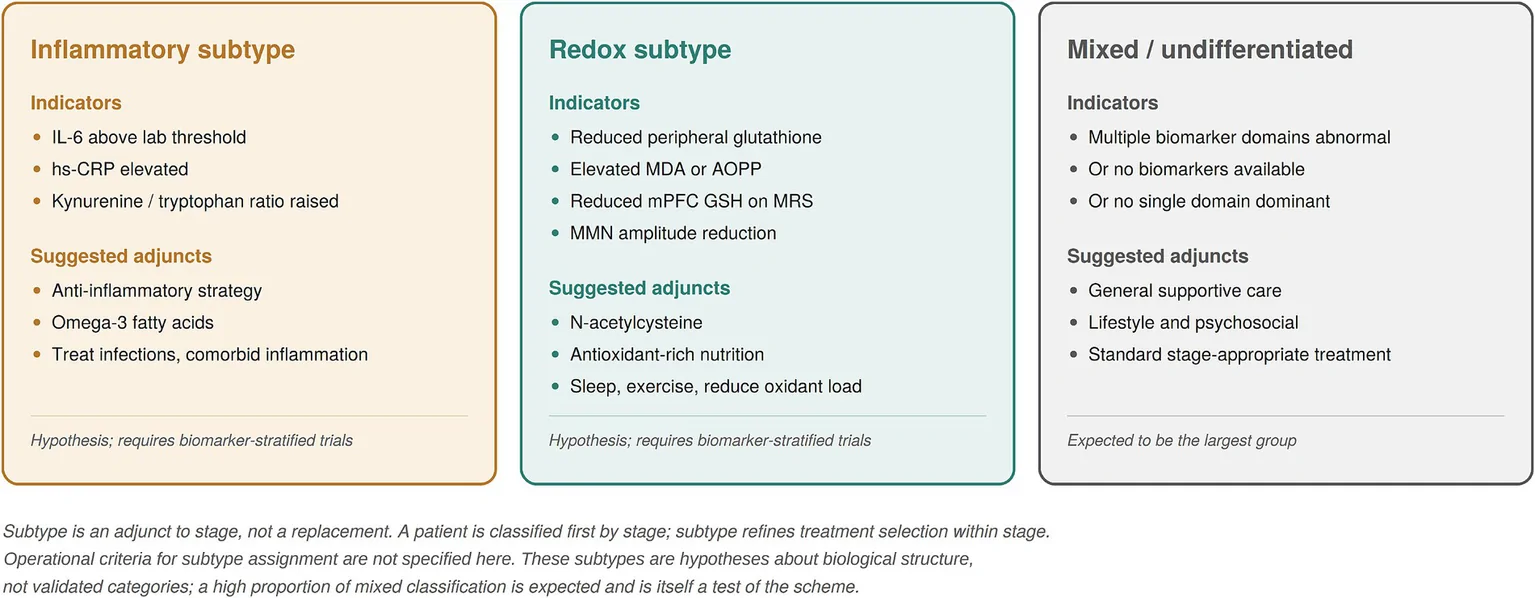
Biomarker subtypes within stage. Patients at the same clinical stage may be biologically different. Three biological subtypes are proposed (inflammatory, redox, mixed), with an undifferentiated category for those not meeting criteria for any; each is associated with a different set of biomarker indicators and a different set of plausible adjunctive interventions. Operational criteria for subtype assignment are not specified here; assignment is proposed to rest on converging indicators rather than on any single assay.

The word *stage* is not used uniformly here, because the boundaries it would span rest on very different amounts of evidence.

Stages 0 and 1 are not disease stages but risk strata: raised vulnerability at population level, with no claim that disease is present in any individual. Stage 0 means documented family history or genomic liability without symptoms; Stage 1 means non-specific symptoms in someone seeking help, most of whom will never develop a psychotic disorder and many of whom will develop an anxiety or mood disorder instead. Stages 2 to 4 describe clinical states with established diagnostic criteria and measurable transition data. The first two levels are therefore renamed Risk Stratum 0 and Risk Stratum 1, and stage terminology reserved for level 2 and above. The five levels are research categories rather than sequential steps through which individuals are expected to pass. Most people in a risk stratum never enter Stage 2, movement between adjacent levels is not assumed to be ordered or irreversible, and an individual may meet criteria for a level without having occupied the one below it.

Neither transition into or out of Risk Stratum 1 has an established probability. No cohort follows people with high liability but no symptoms through to the appearance of non-specific symptoms with schizophrenia-specific outcomes recorded, and the proportion of adolescents with non-specific changes in sleep, mood and thinking who reach an attenuated psychosis syndrome is unknown outside samples already selected for psychosis risk. No figure is given for either, because none would have a primary source.

Stage 2 to Stage 3 is the only boundary with solid data, and even there the numbers are unstable. Across 130 studies and 9,222 people at clinical high risk, cumulative transition risk was 9% at 6 months, 15% at 1 year, 25% at 3 years and 28% beyond 4 years, with between-study heterogeneity of I^2^ 78–96% (Salazar de Pablo et al., 2021); an earlier meta-analysis of 27 studies reported 18%, 22 and 36% at the same intervals (Fusar-Poli et al., 2012). An eleven-point gap at 3 years, between overlapping instruments in overlapping populations, shows what a threshold does. Transition risk at this boundary depends less on the underlying biology than on who is admitted through the door, and it has fallen as clinical high-risk services have widened their intake.

That is the reasoning behind how entry to Risk Stratum 1 should be defined: by persistent non-specific symptoms accompanied by help-seeking, not by the minimal and transient changes common in adolescence, since admitting those would enlarge the stratum without enriching it, which is one mechanism by which reported transition rates fall. Membership does not imply trajectory. Most people in a risk stratum will not develop psychosis.

Whether risk strata belong in a schizophrenia-specific classification depends on use. They belong there for research, to define cohorts for studying a proposed mechanism over time. They do not belong there for clinical classification.

Operational thresholds for these levels and for the biomarker subtypes are deliberately not specified in this article. A draft instrument operationalizing them was generated with the assistance of a large language model during preparation, and its values were not individually adjudicated by the authors. An instrument intended to harmonize classification across research sites should not rest on values of that kind, so it is not presented here (see the statement on the use of artificial intelligence). It will be developed separately, with each threshold individually justified against primary data, and reported together with its psychometric evaluation.

Peripheral BDNF is not proposed as a subtype marker, because it does not distinguish schizophrenia from mood disorders. Mismatch negativity is grouped with the redox subtype, where its link to parvalbumin interneuron function is specified.

The subtypes are hypotheses about biological structure rather than validated categories. Inflammation, redox regulation and circuit function overlap a great deal, so a sizeable proportion of people would be expected to meet criteria for more than one. That is expected, not a fault, and the proportion who cannot be assigned a dominant subtype is itself worth measuring: if most of a cohort cannot be, the scheme has failed and should be abandoned rather than adjusted. One constraint follows from the effect sizes in this literature. No single biomarker should define a subtype on its own. Medial prefrontal glutathione, the most direct available measure of the proposed mechanism, differs between patients and controls by a small standardized margin (Sydnor and Roalf, 2020), and was not reduced in one antipsychotic-naive clinical high-risk sample (Da Silva et al., 2018).

Stage is assigned on clinical phenomenology and biology, not chronological age. A fourteen-year-old can present at Stage 3, as can a fifty-five-year-old, and anchoring stages to age would misclassify both. The typical epidemiological distribution provides orientation only and is given in Supplementary Figure S1, with peak onset at fifteen to twenty-five in males and twenty to twenty-nine in females (Häfner, 2019).

### 4.1 Stage 0: Asymptomatic increased risk

Stage 0 refers to individuals with elevated risk who are clinically asymptomatic. The typical window is childhood and early adolescence in genetically high-risk cohorts (first-degree relatives, 22q11.2 deletion carriers and those with very high polygenic risk), though the stage is age-agnostic. Risk contributors include family history (first-degree relatives confer 5–10-fold population risk), high polygenic risk score (Trubetskoy et al., 2022), high-penetrance CNVs (Marshall et al., 2017), and environmental exposures (perinatal complications, childhood adversity, urbanicity, cannabis; Khandaker et al., 2015; Murray et al., 2017). Symptoms are absent, function preserved, and most biomarkers within population reference ranges. Risk assessment at this stage rests on family history and polygenic risk score, which are the validated genetic measures. Antioxidant-capacity genotype is proposed here as a research stratification variable rather than a screening test: low-capacity GCLC or GCLM variants may mark a subgroup with a reduced ceiling on glutathione synthesis, but the supporting evidence is candidate-gene work in small cohorts, and whether such genotyping adds anything to polygenic risk in this population is unknown and worth testing. Stage 0 carries no indication for antipsychotic treatment. Measures addressing modifiable environmental risk are appropriate on broader grounds, and structured follow-up is reasonable for those with very high genetic vulnerability.

Stratifying asymptomatic children and adolescents by genetic risk raises ethical problems that labeling the work research-only does not remove, and the arithmetic makes them concrete. Against a lifetime base rate near 1%, the five to tenfold relative risk conferred by an affected first-degree relative corresponds to an absolute lifetime risk of roughly 5 to 10%, so most people identified this way will never develop the illness. The same arithmetic applies to polygenic scores: a relative elevation that sounds large translates, against a 1% base rate, into a low absolute risk and a positive predictive value in the low single digits. Identification therefore risks stigma and a self-fulfilling expectation in young people who are overwhelmingly unlikely to become ill, and protections against genetic discrimination vary by jurisdiction and do not reliably extend to every form of insurance. Research at this level should require age-appropriate assent with parental consent, genetic counseling, no return of individual risk estimates outside a protocol designed for it, and data governance that keeps risk classification out of clinical and educational records.

### 4.2 Stage 1: Non-specific symptoms

Stage 1 corresponds to mild cognitive change, sleep disturbance, mood or anxiety symptoms, mild social withdrawal, and subtle motor or academic decline, without attenuated psychotic symptoms. Typical age window for orientation: early-to-middle adolescence (approximately 12–17), reflecting that the prodromal phase typically begins two to 5 years before any psychotic episode; older first presentations occur and are not excluded. The features have low specificity and overlap with adolescent mood and anxiety disorders. Biologically, stage 1 reflects an early redox, inflammatory and synaptic drift in which compensatory systems remain largely intact (Cabungcal et al., 2013; Fraguas et al., 2019; Steullet et al., 2017). Subtle changes in cortical thickness, white-matter integrity and resting-state network organization have been reported in adolescent at-risk samples (Di Camillo et al., 2024). Reduced peripheral BDNF and small reductions in MMN amplitude can precede frank psychotic symptoms (Fernandes et al., 2015; Perrottelli et al., 2021).

Stage 1 interventions should be proportionate and supported by adolescent mental health evidence: treatment of comorbid mood, anxiety and substance-use problems; sleep optimization; structured exercise; family psychoeducation. Pharmacological prevention specific to schizophrenia is not justified at stage 1; antipsychotic exposure carries metabolic and neurological risks without an evidence base for primary prevention. Omega-3 fatty acids and N-acetylcysteine have biological rationales and acceptable safety profiles, but neither has been tested for prevention at this stage, and the largest omega-3 prevention trial, conducted at the next stage, was negative (Section 4.3).

### 4.3 Stage 2: Clinical high risk (CHR/UHR)

Stage 2 corresponds to the CHR/UHR state introduced in Section 2 (Fusar-Poli et al., 2013a; Yung and Nelson, 2011; Yung, 2017). Typical age window for orientation: mid-to-late adolescence and early adulthood (approximately 14–25), the standard recruitment window for CHR services. Transition rates of approximately 20–35% within 2–3 years have been reported across services. Three biomarker domains have unusually strong evidence at this stage: MMN amplitude reduction (Erickson et al., 2016; Hamilton et al., 2022; Tada et al., 2025); presynaptic striatal dopamine synthesis capacity on [18F]-DOPA PET (Howes and Kapur, 2009; McCutcheon et al., 2019); and multivariate MRI signatures (Di Camillo et al., 2024; Kambeitz et al., 2015). Additional signals include reduced medial prefrontal glutathione (Zhang et al., 2025), elevated peripheral cytokines in subgroups (Goldsmith et al., 2016), kynurenine pathway dysregulation (Marx et al., 2021), reduced peripheral BDNF (Fernandes et al., 2015), and polygenic risk score (Trubetskoy et al., 2022).

The heterogeneity of the CHR state is a principal reason that broad interventions tested in unselected CHR cohorts have produced inconsistent results. Defining biomarker-defined subgroups, for example, a high-inflammation, low-glutathione subgroup and matching them to mechanism-appropriate interventions is increasingly seen as the more plausible path to demonstrable disease modification (Fillman et al., 2016; Mongan et al., 2021; Zaki et al., 2022).

Cognitive-behavioral therapy is the best-supported intervention for the CHR state and reduces symptom severity; meta-analyses suggest possible reduction in transition risk, although magnitude and durability are contested (Fusar-Poli et al., 2013a). Antipsychotic medication is not first-line for unselected CHR cases. N-acetylcysteine and anti-inflammatory strategies have plausible biological rationales but inconsistent prevention data in unselected cohorts (Fraguas et al., 2019). For omega-3 fatty acids the record is clearer and less favorable. A single-center trial in 81 ultra-high-risk individuals reported transition in 2 of 41 on omega-3 against 11 of 40 on placebo at 12 months (Amminger et al., 2010), but the multicenter NEURAPRO trial in 304 participants found no difference, with 12-month transition of 11.5% on omega-3 and 11.2% on placebo (McGorry et al., 2017). Its authors noted that a lower than expected transition rate and good psychosocial care in both arms may have limited the test, but concluded that omega-3 is not effective where good quality psychosocial treatment is available. NEURAPRO is consistent with the stratification argument made here, since it enrolled an unselected sample, but it is also the most direct evidence against omega-3 as a general preventive, and it is counted as such. Low-dose lithium has been examined: Berger et al. (2012) reported, in an open-label MRI/MRS study of UHR individuals, that low-dose lithium was associated with apparent preservation of hippocampal microstructure, supporting a brain-level signal in a small uncontrolled sample. Rice et al. (2024) conducted an open-label pilot trial; lithium was well tolerated and transition was numerically lower in the lithium group than in treatment as usual, but the study was not powered to establish efficacy and did not demonstrate a statistically significant reduction. These results demonstrate feasibility and biological plausibility, not prevention; adequately powered biomarker-stratified trials are required.

### 4.4 Stage 3: First-episode psychosis

Stage 3 marks the formal clinical onset and is the point at which schizophrenia (or a schizophrenia-spectrum disorder) becomes diagnosable. Typical age window for orientation: peak first-episode incidence falls at ages 15–25 in males and 20–29 in females, with mean age at first hospitalization of approximately 28 years in males and 32 years in females (Häfner, 2019); approximately 1 % of first episodes occur before age 13 (childhood-onset schizophrenia), and approximately 20 % of first episodes occur after age 40 (late-onset schizophrenia). Stage 3 is therefore the most age-dispersed of the stages and requires explicit awareness that the framework does not exclude early or late presentations. The first-episode phase remains biologically more dynamic than chronic schizophrenia and is therefore a critical window.

Cognitive deficits in verbal memory, executive function, attention, working memory and processing speed are present from the earliest clinical phase and predict subsequent functional outcome more strongly than positive-symptom severity (Aas et al., 2014; Bozikas and Andreou, 2011; Lindgren et al., 2020). Glutathione reductions, elevated cytokines, kynurenine dysregulation, MMN reduction and gray-matter changes are all clearly expressed by this stage (Goldsmith et al., 2016; Guo et al., 2014; Leung et al., 2011; Marx et al., 2021; Perrottelli et al., 2021; Zhang et al., 2025). Multimodal panels distinguish first-episode patients from healthy controls with high accuracy in discovery cohorts (Shen et al., 2024; Zaki et al., 2022).

Antipsychotic medication is the cornerstone of first-episode treatment. D2 antagonism, however, does not address the upstream inflammatory, oxidative, mitochondrial and cognitive abnormalities, and outcomes for negative symptoms and cognition remain unsatisfactory. Several adjunctive approaches are biologically plausible: omega-3 fatty acids, N-acetylcysteine, anti-inflammatory strategies and low-dose lithium adjunctive to antipsychotic. Early consideration of clozapine in indicated cases is supported separately, on efficacy grounds (Section 5.3).

### 4.5 Stage 4: Recurrent, persistent or treatment-resistant

Stage 4 encompasses recurrent psychosis, incomplete remission, severe persistent illness and treatment-resistant schizophrenia. Typical age window for orientation: most patients reach Stage 4 from approximately 5 years after first-episode onset, which places typical Stage 4 presentations in the late twenties onward; the stage extends across mid-adulthood and into older age, with patient cohorts in their forties, fifties and beyond well represented. Stage 4 is characterized by consolidation of the abnormalities seen at first episode: persistent oxidative stress and inflammatory dysregulation (Flatow et al., 2013; Juchnowicz et al., 2021), persistent kynurenine pathway dysregulation in subgroups (Goldsmith et al., 2016; Marx et al., 2021), progressive structural change correlated with relapse number and duration of untreated psychosis, and cognitive impairment that remains the strongest predictor of long-term function (Lindgren et al., 2020). Suicidality, premature cardiovascular and metabolic mortality, and reduced life expectancy are substantial concerns. Despite this consolidation, stage 4 is not synonymous with biological stasis, and opportunities for harm reduction and functional improvement remain.

## 5 Therapeutic implications of the framework

If redox-inflammatory imbalance is an early and modifiable point of entry, the therapeutic question becomes which interventions act on that upstream biology and at which stages. This section addresses the four classes of intervention with the clearest mechanistic case: lithium, N-acetylcysteine and omega-3 fatty acids, clozapine, and repetitive transcranial magnetic stimulation. Evidence is presented with appropriate caution; none of these interventions is currently established as disease-modifying in schizophrenia. Figure 14 shows where each acts on the cascade.

**Figure 14:**
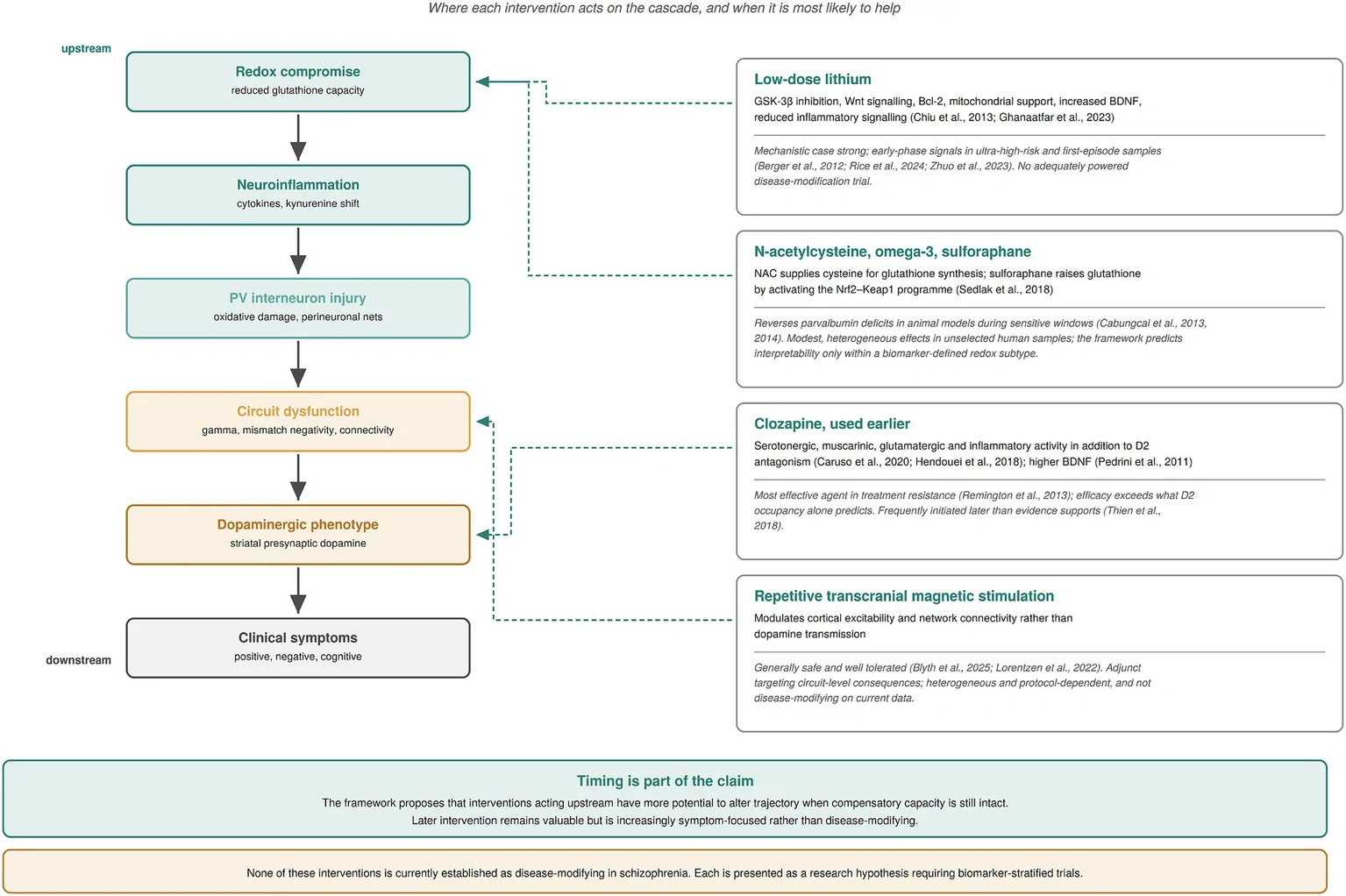
Where the candidate interventions act on the upstream cascade. The cascade runs from redox compromise through neuroinflammation, parvalbumin interneuron injury, circuit dysfunction and the dopaminergic phenotype to clinical symptoms. Low-dose lithium and the glutathione-directed agents act at the upstream end: lithium through glycogen synthase kinase-3β inhibition, mitochondrial support and reduced inflammatory signaling (Chiu et al., 2013; Ghanaatfar et al., 2023), and N-acetylcysteine and sulforaphane by supplying cysteine or activating the Nrf2-Keap1 program (Sedlak et al., 2018). Clozapine and repetitive transcranial magnetic stimulation act downstream, on the dopaminergic phenotype and on circuit-level consequences, respectively, (Caruso et al., 2020; Blyth et al., 2025). Citations for each claim are given in the figure and correspond to the evidence set out in this section. The framework proposes that interventions acting upstream have more potential to alter trajectory while compensatory capacity remains intact, and that later intervention is increasingly symptom-focused. None of these interventions is currently established as disease-modifying in schizophrenia; each is presented as a research hypothesis requiring biomarker-stratified trials.

### 5.1 Low-dose lithium

Lithium acts at several points upstream of the dopaminergic phenotype that D2 antagonists target: it inhibits glycogen synthase kinase-3β, upregulates Wnt/β-catenin signaling, increases Bcl-2 expression, supports mitochondrial function, increases BDNF and reduces inflammatory signaling (Chiu et al., 2013; Ghanaatfar et al., 2023; Puglisi-Allegra et al., 2021). In animal models it reduces oxidative damage, supports parvalbumin interneuron survival under oxidative challenge, and improves cognitive outcomes. Figure 14 places these actions on the cascade.

The clinical evidence is early. An open-label MRI/MRS study reported apparent preservation of hippocampal microstructure with low-dose lithium, in a small uncontrolled sample (Berger et al., 2012). A trial in ultra-high-risk youth found low-dose lithium well tolerated, with numerically lower transition than treatment as usual, but was not powered for efficacy (Rice et al., 2024). Adjunctive low-dose lithium in first-episode patients has shown signals on inflammation, gray-matter trajectory and cognition in small studies, consistent with disease modification rather than symptom suppression (Zhuo et al., 2023). Ecological observations linking lithium in drinking water to lower rates of psychiatric illness and suicide (Kessing et al., 2017; Shimodera et al., 2018) are hypothesis-generating only, since confounding and inconsistent replication preclude causal inference.

The position is therefore a strong mechanistic case, encouraging early-phase signals, and no definitive disease-modification data. A biomarker-stratified randomized trial in the redox subtype is the test lithium most needs.

### 5.2 N-acetylcysteine, sulforaphane, and omega-3 fatty acids

N-acetylcysteine supplies cysteine for glutathione synthesis and scavenges reactive oxygen species directly. It reverses parvalbumin interneuron deficits induced by developmental oxidative challenge in animal models (Cabungcal et al., 2013; Cabungcal et al., 2014), but in adults with schizophrenia the signals on negative symptoms and cognition are modest and heterogeneous, and in clinical high-risk populations the evidence base is too small for firm conclusions. Sulforaphane raises glutathione by a different route, activating the Nrf2-Keap1 program rather than supplying substrate; a clinical pilot found short-term oral administration increased peripheral and brain glutathione in humans (Sedlak et al., 2018), while randomized trials in schizophrenia report inconsistent effects. Omega-3 polyunsaturated fatty acids reduce inflammation, support membrane integrity and modulate BDNF expression.

One prediction applies across all three, and it has to be stated in a form that a null result can contradict. The framework proposes that an antioxidant given to someone without redox compromise has nothing to correct, so benefit in an unselected trial should be diluted in proportion to the share of participants with the relevant subtype. The broader argument, that antioxidant trials in central nervous system disease have largely enrolled unselected samples and measured damage rather than regulatory state, is developed in Lado (2026). That dilution argument is a claim, not an escape. Suppose the redox subtype makes up a fraction f of an unselected sample, and the framework predicts an absolute reduction d in the outcome within it. The unselected trial should then show an effect of roughly f times d. An unselected trial precise enough to exclude an effect of that size, with f estimated from baseline biomarkers in the same participants, counts against the framework before any stratified trial is run; so does a trial in which benefit does not increase with baseline redox compromise. To put a magnitude on the rule: if a fifth of an unselected sample has the redox subtype and the framework predicts a reduction of ten percentage points in transition within it, the unselected trial should show a reduction of about two percentage points, and a trial precise enough to exclude a benefit of two points counts against the framework. Interpretable trials still require biomarker-defined subgroups, redox for the first two agents and inflammatory for omega-3, but null results in unselected samples are not treated here as expected by default.

### 5.3 Clozapine, used earlier

Clozapine is the most effective antipsychotic in treatment-resistant schizophrenia, with a particular role in reducing suicidality and hospitalization (Remington et al., 2013). Its efficacy exceeds what D2 occupancy alone predicts, and it carries relevant activity at serotonergic, muscarinic, histaminergic, glutamatergic and inflammatory targets (Caruso et al., 2020; Hendouei et al., 2018), with BDNF positively associated with exposure (Pedrini et al., 2011). The framework does not claim those actions as a mechanism. On its own logic, clozapine should be expected to burden the upstream node rather than relieve it: it causes weight gain and metabolic syndrome, has been associated with inflammatory activation, and its metabolism can generate reactive species. The case for earlier use made here therefore rests on efficacy alone.

Clozapine is initiated later than the evidence supports (Thien et al., 2018). In the only randomized study to address sequencing, first-episode patients who had not remitted on amisulpride did no better when switched to olanzapine than when continued on amisulpride (44% versus 45% remission), and the investigators concluded that clozapine should follow the first failed antipsychotic trial rather than the second (Kahn et al., 2018); its open-label clozapine phase was small and brief, however, and remission in that phase was modest. In a first-episode algorithm, response fell from 75% on the first antipsychotic to 17% on the second, then returned to 75% on clozapine (Agid et al., 2011). Longer delay before clozapine has been associated with poorer response in treatment-resistant schizophrenia (Üçok et al., 2015; Yoshimura et al., 2017), and a meta-analysis found a small advantage for clozapine used first or second line (Okhuijsen-Pfeifer et al., 2018). The delay studies are retrospective and share the confounding by indication noted in Section 3.8, since patients who reach clozapine late may have been more severely ill from the outset. No trial has randomized patients to early versus delayed clozapine. Earlier consideration in first-episode patients with poor response, severe symptoms, persistent suicidality or rapid deterioration follows from the efficacy data. It also yields a prediction the framework can be tested against: if clozapine’s metabolic and inflammatory burden worsens the proposed upstream biology, early users should show adverse change in redox and inflammatory markers despite clinical improvement, and those markers warrant monitoring wherever clozapine is used early. The United States Food and Drug Administration eliminated the clozapine Risk Evaluation and Mitigation Strategy in 2025, ending mandatory reporting of absolute neutrophil counts and the certification requirements attached to dispensing. Monitoring was not withdrawn: the agency continues to recommend absolute neutrophil count monitoring at the frequencies given in the prescribing information, and the boxed warning for severe neutropenia remains. The administrative barrier fell, not the clinical vigilance.

### 5.4 Repetitive transcranial magnetic stimulation

Repetitive transcranial magnetic stimulation and intermittent theta-burst stimulation have been evaluated as adjuncts for negative symptoms, cognitive deficits and treatment-resistant auditory hallucinations. Evidence is heterogeneous and protocol-dependent, though the technique is generally safe and well tolerated in schizophrenia-spectrum illness without clear increase in seizure risk (Blyth et al., 2025; Lorentzen et al., 2022). It modulates cortical excitability and network connectivity rather than dopamine transmission, which places it in this framework as an adjunct targeting circuit-level consequences of upstream pathology rather than as a disease-modifying treatment.

### 5.5 What about emerging targets?

Several emerging therapeutic targets deserve mention. Trace amine-associated receptor 1 (TAAR1) agonists were proposed as a non-D2 mechanism for psychosis treatment (McCutcheon et al., 2020); ulotaront, the first TAAR1 agonist to reach late-phase trials, did not separate from placebo in its pivotal phase 3 studies, and the place of TAAR1 agonism in the treatment landscape remains uncertain. Muscarinic M1/M4 agonists, of which xanomeline-trospium (KarXT) achieved FDA approval in 2024, target a different non-D2 mechanism and represent the first new mechanism class of antipsychotic in decades. Glucagon-like peptide-1 receptor agonists, with metabolic and possible neuroinflammatory effects, are being explored. Each of these targets sits closer to downstream symptom management than upstream disease modification; their inclusion in the framework is appropriate at stages 3 and 4 rather than as primary prevention candidates.

## 6 Falsifiable predictions and a biomarker-stratified trial design

The framework yields three predictions specific enough to be refuted. The first tests the central therapeutic claim, that matching treatment to biological subtype outperforms treating everyone alike. The second tests the proposed mechanism, that upstream brain markers predict transition better than clinical assessment alone. The third tests whether the redox measures are doing the predictive work rather than traveling alongside markers that are. Together they ask whether the upstream biology is real, whether it can be measured, and whether acting on it changes what happens to patients. If they hold, the framework gains support; if they fail, it must be revised or abandoned.

Table 1 summarizes the three predictions, the part of the argument each tests, and the result that would count against the framework.

**Table 1:** The three falsifiable predictions of the framework, the claim each tests, and the result that would count as evidence against the framework.

Prediction	What it claims	What it tests	What would refute it
1. Stratified prevention	Matching treatment to a person’s biological subtype works better than giving everyone the same treatment.	The central therapeutic claim: upstream biology is the right treatment target.	Subtype-matched treatment does no better than unmatched treatment.
2. Brain markers add predictive value	Adding two upstream brain markers, low glutathione and an abnormal EEG signal, to clinical assessment improves prediction of who develops psychosis.	The proposed mechanism: the redox–NMDA–circuit chain.	The brain markers add nothing beyond clinical predictors.
3. Redox components contribute independently	The redox measures within a multimodal panel contribute independently: removing them worsens prediction.	The premise that redox biology is doing the predictive work.	Removing the redox measures does not worsen prediction.

### 6.1 Prediction 1: Biomarker-stratified prevention outperforms unselected prevention

Stage 2 (CHR/UHR) individuals classified as inflammatory subtype or redox subtype by biomarker panel will show greater benefit from mechanism-matched adjunctive intervention (anti-inflammatory strategies, NAC, omega-3 fatty acids, low-dose lithium for the redox subtype) than from non-matched intervention or treatment as usual. This is falsifiable by a biomarker-stratified randomized trial in CHR cohorts. If stratification does not produce differential response, the framework is weakened, and the case for biomarker subtyping at this stage is weakened.

### 6.2 Prediction 2: Upstream brain markers add predictive value beyond clinical assessment

Clinical high risk is identified with one of two semi-structured interviews: the Comprehensive Assessment of At-Risk Mental States (CAARMS) or the Structured Interview for Psychosis-Risk Syndromes (SIPS). Both define risk by attenuated positive symptoms, brief intermittent psychosis, or genetic risk accompanied by functional decline. In Stage 2 cohorts, adding reduced medial prefrontal glutathione on magnetic resonance spectroscopy and mismatch negativity amplitude deficit to these clinical predictors, together with functional decline and family history, will improve prediction of transition to Stage 3 beyond the clinical predictors alone. The test is the change in area under the curve when the two biological measures are added to a model already containing the clinical instrument, not a head-to-head comparison: Stage 2 is defined by the same attenuated-symptom criteria that CAARMS and SIPS use, so a direct comparison would be circular. If the biological measures add no incremental value, the proposed link from antioxidant depletion through impaired NMDA-dependent signaling to transition is not supported in that dataset.

### 6.3 Prediction 3: The redox components of a multimodal panel contribute independently

In Stage 2 cohorts, a multimodal panel combining polygenic risk score, structural and functional MRI features, mismatch negativity, and peripheral inflammatory and redox markers will predict transition more accurately than the same panel with the redox measures removed. This replaces the broader claim that a multimodal panel outperforms polygenic risk score alone, which is too unspecific to test this framework: a large panel could beat a genetic score for reasons unconnected to redox biology. Removing the redox components isolates their contribution. If prediction does not deteriorate when they are removed, the framework’s central premise is not supported in that dataset.

### 6.4 A schematic trial design

A design in which each biomarker subtype receives a different matched treatment cannot be interpreted, because subtype and treatment are confounded: any difference in outcome could come from the treatment, from the subtype, or from the combination, with no way to separate them.

The design proposed here measures biomarker subtype at baseline in everyone and randomizes to treatment within each subtype, with each treatment appearing in more than one subtype. That separates the effect of a treatment from the effect of matching it to biology. The primary analysis is the treatment-by-subtype interaction, specified in advance: for the framework to be supported, that interaction must be significant. Improvement within a subtype is not sufficient, since people improve for reasons unrelated to biomarker matching.

In practice, Stage 2 individuals identified by CAARMS or SIPS would undergo baseline assessment with a multimodal panel spanning the three domains, circuit-level, redox and inflammatory, then be stratified and randomized within stratum to cognitive-behavioral therapy with or without a mechanism-matched adjunct. Smoking status and cigarette consumption, body mass index, diet and physical activity are recorded at baseline and at each follow-up and entered as covariates, for the reasons given in Section 3.7.

Transition should not be the primary outcome of a first trial, because the sample it requires is prohibitive. Interaction effects need several times the sample of main effects, and transition is uncommon. Taking the pre-specified primary contrast as the redox subtype against the remaining participants, and assuming that transition falls from 20 to 10% with the matched adjunct in the redox subtype and is unchanged elsewhere, detecting the treatment-by-subtype interaction on the log-odds scale with 80% power at a two-sided alpha of 0.05 requires about 357 participants in each of the four subtype-by-arm cells, roughly 1,400 in total before attrition. The design proposed here is therefore a phase 2b trial with a continuous primary outcome: change in medial prefrontal glutathione on magnetic resonance spectroscopy, with change in mismatch negativity amplitude as the key secondary biological outcome and transition as a secondary clinical outcome. For a standardized interaction effect of 0.5, meaning that the adjunct’s effect on the continuous outcome differs by half a standard deviation between subtypes, the same power and alpha require 126 participants per cell, 504 in total, or about 600 allowing for 15% attrition. An interaction of that size is an assumption rather than an estimate, and the calculation should be repeated once pilot data give the variance of change scores. Both calculations are derived in the Supplementary Statistical Note. A positive phase 2b result would justify a transition-powered trial; a null one would count against Prediction 1 at far lower cost. The practical difference for a clinical service is large. A transition-powered trial of roughly 1,400 participants is beyond what any single early intervention service can recruit and would take years to read out, whereas 600 is within reach of a small consortium. It also asks the questions in the right order: whether the intervention moves the biology it is meant to move, and whether it moves it more in the subtype selected for that abnormality, before anyone commits to a trial powered on psychosis itself.

An operational staging instrument is not presented here, for the reasons given in Section 4, and will be developed and evaluated separately. The trial design does not depend on it: stratification requires only biomarker criteria fixed in the trial protocol before enrollment.

## 7 Discussion

### 7.1 What this framework establishes

The contribution of this manuscript is integrative. The clinical staging structure originates with McGorry and colleagues (McGorry et al., 2018; McGorry et al., 2006; McGorry and Hickie, 2019). The neuroprogression formulation originates with Berk and colleagues (Berk et al., 2011; Davis et al., 2016). The biomarker findings are drawn from a literature that long predates this manuscript and that has been ably synthesized elsewhere (Lozupone et al., 2017; Sierakowska et al., 2025; Zaki et al., 2022; Yue et al., 2022). What the present manuscript attempts is to bring these traditions together explicitly under a single thesis, that redox-inflammatory imbalance is an early and modifiable point of entry into the process that produces schizophrenia, and to articulate the testable predictions and trial design that follow from that thesis.

The framework supports three specific claims. First, the biological processes most consistently identified in schizophrenia are mechanistically interconnected, with oxidative stress functioning as an integrative node connecting genetic, developmental, inflammatory, mitochondrial and neuroplastic biology. Second, individuals meeting the same clinical-stage criteria are biologically heterogeneous, and biomarker-based subtyping within stage is likely to be more useful than diagnostic category alone for selecting interventions. Third, the most plausible adjunctive interventions (low-dose lithium, NAC, omega-3 fatty acids and anti-inflammatory strategies) act on upstream biology that becomes less modifiable as the disease consolidates. Earlier clozapine in indicated cases is recommended on efficacy grounds alone, not as an upstream intervention (Section 5.3).

### 7.2 What this framework does not establish

Three claims are worth ruling out explicitly. First, the framework does not claim that schizophrenia can be prevented. No intervention has been shown in adequately powered trials to prevent transition from CHR to first-episode psychosis; the most promising signals (low-dose lithium in pilot data, omega-3 in earlier UHR work, NAC, CBT-CHR) are preliminary and inconsistent. The framework provides a structured way to test prevention hypotheses; it does not prove them. Second, it does not claim that D2 antagonism should be displaced as first-line treatment for first-episode psychosis. Antipsychotic medication remains the cornerstone of treatment at stage 3 on present evidence; what the framework suggests is that the upstream biology represents an underexploited target for adjunctive intervention. Third, it does not claim that the biomarker panels described here are ready for routine clinical use. Any thresholds eventually proposed for them will require empirical calibration against local laboratory reference ranges and psychiatric comparison cohorts before clinical deployment.

### 7.3 On heterogeneity of course

Schizophrenia does not follow one course. A substantial minority have a single episode and recover well; others relapse and remit; others decline slowly and continuously. Any framework proposing a progressive mechanism has to explain why the same biology produces such different outcomes, and whether the most advanced stage is unavoidable.

Both answers turn on the same quantity. Course is set here not by whether redox compromise is present, but by the balance between oxidative burden and antioxidant reserve, meaning how much the defense system can absorb over time (Lado, 2026). Reserve varies with glutathione-synthesis genotype, hormonal status, the developmental stage at which an insult occurs, and accumulated environmental load. Two people carrying the same burden but different reserve follow different courses: a single episode with good recovery reflects temporary burden against intact reserve, a relapsing course reflects intermittent burden against partly reduced reserve, and continuous decline reflects sustained burden against depleted reserve. The most advanced stage is one possible endpoint, not a destination everyone reaches.

Preliminary support comes from how different markers behave across illness phase. A systematic review of twenty-two studies found superoxide dismutase activity and total antioxidant capacity higher, and advanced oxidation protein products lower, in remission than in acute phases, while erythrocyte superoxide dismutase activity stayed altered regardless of clinical state (Flores et al., 2026). That is what the reserve account predicts: markers of current burden should track clinical state, markers of underlying capacity should not. These are cross-sectional comparisons and do not show that reserve determines course, only that the two kinds of marker behave differently.

The account is testable. Baseline measures of reserve, including GCLC and GCLM genotype and glutathione measured peripherally or centrally, should predict course independently of stage at presentation. If course proves unrelated to baseline reserve in adequately powered longitudinal cohorts, the framework loses its main explanation for why outcomes vary so widely under a single proposed mechanism. It also explains why staging is paired here with biomarker stratification rather than used alone: two people at the same stage may face different prognoses if their reserve differs.

### 7.4 Limitations

A further question is whether the progression described here can be told apart from normal aging. Many of the markers discussed (reduced glutathione, inefficient mitochondria, raised inflammatory markers and cortical thinning) also change with age in healthy people, and in the same direction. Recent work makes the overlap explicit, proposing oxidative stress and chronic low-grade inflammation as the hub connecting epigenetic age acceleration, telomere shortening and the senescence-associated secretory phenotype in schizophrenia (Ge et al., 2026).

The question is old: Kraepelin’s *dementia praecox* claimed exactly this, a deteriorating course resembling senile decline arriving early. The account here differs. Cognitive impairment in schizophrenia is largely established by the first years of illness and does not follow the neuropathological course of the dementias (Zipursky et al., 2013), which is why the process proposed here is located in the years before and around onset rather than across the lifetime (Section 3.8). What is proposed is a failure of homeostatic resistance, which is a different kind of process from either dementia or aging, and three features separate it from normal aging. Rate: these changes occur over years to a decade rather than decades, concentrated within a developmental window. Location: age-related redox change is relatively diffuse, whereas this pattern converges on parvalbumin-expressing interneurons and gamma-generating circuitry, a cell population whose metabolic demand makes it particularly vulnerable. Compensation: in healthy aging antioxidant and mitochondrial compensation remain broadly intact and decline is buffered, whereas this framework proposes those systems are themselves compromised, so that a burden a healthy system would absorb instead spreads.

The distinction is testable. The framework predicts that the *rate* of biomarker change, rather than the level at any timepoint, should separate schizophrenia from age-matched controls, and that compensatory capacity should be measurably reduced independently of oxidative burden. Neither can be assessed on current evidence, which is overwhelmingly cross-sectional, a limitation acknowledged within the accelerated-aging literature itself (Ge et al., 2026). The same limitation applies to the comparison between drug-naive first-episode and longer-duration cohorts: cross-sectional differences between those groups cannot distinguish abnormalities established before onset from change accumulating after it. What would separate the two accounts is longitudinal measurement within individuals, and no such data exist at adequate scale.

Structural limitations affect the literature this framework rests on. Most biomarker studies compare patients to healthy controls rather than to bipolar disorder or psychotic depression, so disorder-specific signatures are not established. Antipsychotic exposure modulates dopamine, glutamate, inflammation and the gut microbiome, making cross-sectional findings in treated patients difficult to interpret causally. Most cohorts are European-ancestry adults, and performance in adolescent, multi-ancestry and primary-care populations is unknown (Lozupone et al., 2017). Several of the most promising modalities (PET, magnetic resonance spectroscopy and multimodal MRI) are not feasible for routine use.

The framework has its own limits. The staging structure imposes discrete categories on what is almost certainly a continuous process, and classification at the margins is unreliable. The biomarker subtypes are provisional and may not survive contact with data. The therapeutic recommendations are research framings, not clinical prescriptions.

### 7.5 The path forward

The most important next step is empirical. The staging and subtype structure described here requires operational criteria that are individually justified against primary data and then evaluated for inter-rater and test–retest reliability, concurrent validity against CAARMS, SIPS, DSM-5 and ICD-11, predictive validity in clinical high-risk cohorts, and utility within biomarker-stratified trials. That work will be reported separately.

The field requires the trials that this framework predicts. The phase 2b design in Section 6 is feasible within existing multi-site consortia with spectroscopy capacity. The biomarker-stratified first-episode trial testing whether adjunctive NAC, omega-3 or low-dose lithium in the redox or inflammatory subtype produces measurable change in upstream biology and downstream outcomes is similarly within reach. Without such trials, the framework remains a useful organizing structure that cannot yet claim to alter treatment. With them, it could change what we do at the bedside.

## 8 Conclusion

This manuscript has argued that schizophrenia may be better understood not as a static neurotransmitter disorder but as a developmental process, concentrated in the years before and around onset, in which redox-inflammatory imbalance offers an early and modifiable point of entry into the cellular, circuit and neurotransmitter abnormalities that produce clinical symptoms. The human evidence for that position establishes association and biological plausibility; the causal direction remains to be demonstrated. The staging framework of McGorry and the neuroprogression framework of Berk provide complementary scaffolding for this view, and the biomarker literature provides increasingly specific tools for measurement. The integration set out here yields three falsifiable predictions and a trial design that could refute them.

If those trials confirm that biomarker-stratified early intervention alters trajectory, the field will have a meaningfully different treatment landscape than the dopamine-centered one inherited from the twentieth century. If they refute the framework, it should be modified accordingly. Either outcome advances the field, and our aim has been to make the test as explicit as possible. Reframing schizophrenia as modifiable early and partly reversible opens a path toward treatments aimed at the biology rather than only its downstream symptoms, and toward a future in which the trajectory of this illness is not assumed to be fixed.

A final observation reaches beyond schizophrenia. Oxidative stress is implicated across many chronic diseases, and in most it is studied as a correlate: measured once, compared against controls, interpreted as evidence of damage already done. What is rarely asked is whether these conditions have a natural history driven by that biology, whether they can be staged, and whether the point at which compensation fails can be identified before it does. If examining schizophrenia this way proves productive, the approach may generalize: not the specific markers or thresholds, which are disorder-specific, but the discipline of treating redox compromise as a process with a trajectory rather than a finding with a *p*-value. That is a claim for other investigators to test in other diseases, offered as a way of thinking rather than as a result.

## Data Availability

The original contributions presented in the study are included in the article/Supplementary material, further inquiries can be directed to the corresponding author.

## Author’s note

LL dedicates this work to his first-born son, Leonardo. He suffered a great deal, he taught me more than I can express, and he is the reason I believe that there is hope after all.
