# Supplementary material for: Redox compromise as an early, modifiable entry point in schizophrenia: a staging framework for early intervention
Source: Front Neurosci. 2026 Oct 15;20:1941148. doi: 10.3389/fnins.2026.1941148 (PMC13716746; doi:10.3389/fnins.2026.1941148)
Supplement: Supplementary file 1 [file Data_Sheet_1.DOCX]

**Supplementary Figure S1**

**Manuscript 1941148** — Schizophrenia as a Progressive Disease Driven by Upstream Oxidative Stress: A Staging Framework for Early, Disease-Modifying Intervention

**Supplementary Figure S1. Typical age distribution by stage (orientation only — age is not a staging criterion).**

Stage in this framework is assigned on clinical phenomenology and biology, not on chronological age. A fourteen-year-old can present at Stage 3, as can a fifty-five-year-old, and anchoring stages to age would misclassify both. This figure gives the typical epidemiological distribution for orientation only, with peak onset at fifteen to twenty-five years in males and twenty to twenty-nine years in females (Häfner, 2019).

The figure is provided as supplementary material rather than in the main text because it carries no staging criterion and is not used in classification.

**Reference**

Häfner, H. (2019). From onset and prodromal stage to a life-long course of schizophrenia and its symptom dimensions: how sex, age, and other risk factors influence incidence and course of illness. *Psychiatry J.* 2019, 9804836. doi:10.1155/2019/9804836
