# Supplementary material for: Redox compromise as an early, modifiable entry point in schizophrenia: a staging framework for early intervention
Source: Front Neurosci. 2026 Oct 15;20:1941148. doi: 10.3389/fnins.2026.1941148 (PMC13716746; doi:10.3389/fnins.2026.1941148)
Supplement: Supplementary file 2 [file Data_Sheet_2.DOCX]

# Supplementary Evidence Review

Manuscript 1941148 — *Schizophrenia as a Progressive Disease Driven by Upstream Oxidative Stress*

Figure numbers cited in this review refer to figures in the main manuscript. This review presents the biomarker evidence supporting each component of the integrative node described in Section 3 of the main text. Every study referenced here is tabulated in Supplementary Table S1 with its study design, illness stage, treatment status, biomarker source, direction of finding and principal limitation. Evidence tiers follow the hierarchy defined in Section 2.1 of the main text: established, model-supported, or extrapolated.

## S1. Redox biology and its measurement

Three lines of evidence link redox compromise specifically to schizophrenia. Proton magnetic resonance spectroscopy demonstrates reduced glutathione in medial prefrontal cortex (Das et al., 2019), present before extensive antipsychotic exposure, observed across the schizophrenia spectrum, and correlated with clinical phenotype and cognitive performance in several cohorts. Genetic variation in the rate-limiting enzymes of glutathione synthesis, the catalytic (GCLC) and modulator (GCLM) subunits of glutamate-cysteine ligase, has been associated with schizophrenia and with patterns of brain structure, function and peripheral oxidative-stress markers consistent with redox compromise (Gysin et al., 2007; Tosic et al., 2006); Figure 3 summarizes this pathway. Peripheral damage markers including malondialdehyde, advanced oxidation protein products, protein carbonyls and advanced glycation end-products are reliably elevated, and antioxidant enzyme activities are altered in patient samples (Ben Othmen et al., 2008; Flatow et al., 2013; Kulaksizoglu and Kulaksizoglu, 2016; Juchnowicz et al., 2021; Mohib et al., 2025). A recent cross-species study reinforces this picture: antipsychotic-naive patients show elevated serum malondialdehyde and nitric oxide alongside reduced catalase activity, and re-analysis of single-cell RNA-sequencing from the Setd1a⁺/− mouse prefrontal cortex shows reduced expression of *Gpx4* and *Nfe2l2* (Nrf2), connecting the peripheral signature to cell-type-specific transcriptional compromise of the antioxidant defense network (Mohib et al., 2025).

Discriminatory accuracy has been notable: a combination of total oxidant status and glutathione peroxidase has reached approximately 89% accuracy distinguishing patients from healthy controls (Juchnowicz et al., 2021). These results require external validation in psychiatric comparison cohorts before clinical translation.

One functional marker at the intersection of membrane-lipid and redox biology deserves mention. Topical niacin normally provokes flushing through GPR109A-mediated phospholipase-A2 release of arachidonic acid; in a subgroup of patients this flush is blunted, implicating the membrane-lipid compartment vulnerable to oxidative damage (Messamore et al., 2010). Its disorder-relative specificity is of interest: the blunted response is more prevalent in schizophrenia than in bipolar disorder or major depression, is heritable within affected families, and has been proposed as a candidate endophenotype rather than a state marker (Yao et al., 2016). Sensitivity is its principal limitation; it marks a physiologically distinct subgroup rather than serving as a universal test.

## S2. Inflammation and the kynurenine pathway

Meta-analytic evidence demonstrates elevated pro-inflammatory cytokines — IL-6, TNF-α, IL-1β, IL-8, IFN-γ — particularly in the first episode (Goldsmith et al., 2016; Khandaker et al., 2015). High-sensitivity C-reactive protein is elevated in a subset and predicts poorer cognition. The specificity of single cytokines is limited and overlap with depression and bipolar disorder is substantial, but inflammatory subgroups within schizophrenia are increasingly recognized as clinically meaningful and may respond differentially to anti-inflammatory adjuncts (Fillman et al., 2016). Circulating complement component 4, whose gene is among the strongest schizophrenia risk loci through its role in synaptic pruning, is elevated at the protein level in first-episode patients, and a three-marker panel combining endotoxin, HMGB1 and C4 distinguished first-episode schizophrenia from healthy controls with an area under the curve of 0.933 (Chen et al., 2022); specificity against other psychotic disorders remains to be established.

Pro-inflammatory cytokines upregulate indoleamine 2,3-dioxygenase and tryptophan 2,3-dioxygenase, shunting tryptophan from serotonin synthesis into the kynurenine pathway. Kynurenic acid (an NMDA antagonist) and quinolinic acid (an NMDA agonist and excitotoxin) are dysregulated in opposite directions in different brain regions (Cao et al., 2021; Marx et al., 2021; Plitman et al., 2017); elevated CSF kynurenic acid is replicated but shared with bipolar disorder rather than specific to schizophrenia, and a meta-analysis of 101 studies confirmed pathway dysregulation with reductions in plasma kynurenine (Marx et al., 2021). The connection to redox biology is bidirectional: kynurenine pathway dysregulation amplifies oxidative stress, and oxidative stress amplifies the inflammatory state that drives it. A systematic review found elevated IL-6 and TNF-α most consistently associated with kynurenine pathway changes, with IL-8 and lower IL-4 implicated in fewer studies and no significant association for IFN-γ or CRP (Pedraz-Petrozzi et al., 2020). Figure 4 illustrates the branch point.

## S3. Circuit-level readouts

Two classes of measurement index the circuit-level consequences of this biology.

Univariate structural MRI findings are highly replicated but lack individual-level diagnostic specificity. Multivariate pattern recognition reaches pooled sensitivity and specificity of approximately 80% (Kambeitz et al., 2015), a ceiling reproduced across modalities with disease stage, antipsychotic exposure and methodological choices as principal moderators (Di Camillo et al., 2024); in CHR samples, multimodal MRI signatures distinguish those who transition at accuracies of 70–85%, exceeding any clinical predictor alone (Di Camillo et al., 2024). Proton MRS provides in vivo neurochemistry, with elevated glutamate across basal ganglia, frontal and medial prefrontal cortex in the largest synthesis to date (Lopes et al., 2024) and reduced glutathione in first-episode samples (Sydnor and Roalf, 2020). GABA reductions in midcingulate cortex have been reported in first-episode psychosis (McCutcheon et al., 2020). Figure 7 summarizes the regions with reproducible findings. The retina, the only directly imageable CNS tissue, shows consistent thinning of the peripapillary retinal nerve fiber layer and macular ganglion-cell layers across 65 studies, associated with illness duration and symptom severity (Komatsu et al., 2024); retinal measures remain non-specific and are confounded by diabetes and hypertension.

Mismatch negativity is among the most replicated electrophysiological findings, with the largest effect size among event-related potential candidates (Erickson et al., 2016; Tada et al., 2025). It is generated by NMDA-receptor-dependent mechanisms and replicated in animal models, connecting it to the circuit-level substrate described above. MMN amplitude deficits are associated with future transition among CHR individuals, although associations are modest and predictive value is limited alone (Hamilton et al., 2022). Disorder-relative specificity is comparatively favorable: deficits are smaller in bipolar disorder (Erickson et al., 2016; Tada et al., 2025). A systematic review of EEG measures in at-risk and early schizophrenia identified reduced MMN and P300 amplitudes and impaired sensory gating as the most consistent abnormalities (Perrottelli et al., 2021). Figure 8 illustrates the gamma reduction and phase jitter linking the electrophysiological signal to the parvalbumin substrate.

## S4. Secondary domains: neurotrophic, proteomic and microbiome

Metabolomic reviews report reproducible shifts in lipid, amino-acid and energy metabolites at onset, several redox- and glutamate-related (Yao et al., 2025; Davison et al., 2018), and plasma proteomic models have reached AUCs above 0.97 in discovery cohorts (Shen et al., 2024). Metagenome-wide association studies identify reproducible differences in gut microbial composition, with *Streptococcus* repeatedly implicated and alterations in short-chain fatty acid synthesis, tryptophan metabolism and GABA/glutamate synthesis modules (Zhu et al., 2020); integrated models distinguish patients from controls with AUC up to 0.94 in discovery cohorts (Huang et al., 2026). Fecal microbiota transfer from patients into germ-free mice induces schizophrenia-relevant behavioral and neurochemical changes (Zheng et al., 2019), suggesting causal contribution rather than only association. Specificity against depression and bipolar disorder remains incomplete for both domains, and neither is a clinical assay.

## Figures referenced in this review

All figures are in the main manuscript. Figure 3, glutathione synthesis and the GCLC/GCLM bottleneck. Figure 4, the kynurenine pathway. Figure 5, mitochondrial electron transport and complex I. Figure 6, parvalbumin interneurons. Figure 7, brain regions with reproducible findings. Figure 8, auditory electrophysiology. Figure 10, maternal immune activation.
