# Supplementary material for: Redox compromise as an early, modifiable entry point in schizophrenia: a staging framework for early intervention
Source: Front Neurosci. 2026 Oct 15;20:1941148. doi: 10.3389/fnins.2026.1941148 (PMC13716746; doi:10.3389/fnins.2026.1941148)
Supplement: Supplementary file 3 [file Data_Sheet_3.DOCX]

**Supplementary Table S1**

**Characteristics and limitations of the evidence cited in support of the staging framework**

This manuscript is a Hypothesis and Theory article rather than a systematic review, and the evidence assembled here was selected to test the coherence of a proposed framework rather than to survey the field exhaustively. Sources were identified through PubMed and Google Scholar using terms covering oxidative stress, redox regulation, glutathione, neuroinflammation, clinical staging and neuroprogression in schizophrenia and psychosis, with priority given to meta-analyses, longitudinal cohorts and randomized trials where available. This table records, for each body of evidence cited in Sections 3 and 4, the study design, illness stage, treatment status, biomarker source, direction of finding and principal limitation. Null and contradictory findings are reported alongside supportive ones. Entries are grouped by biomarker domain and, within each domain, follow the order of presentation in the main text.

| **Study** | **Design** | **Illness stage** | **Treatment status** | **Biomarker source** | **Direction of finding** | **Principal limitation** |
| --- | --- | --- | --- | --- | --- | --- |
| **A. Redox status — central measures** | | | | | | |
| Das et al., 2019 | Meta-analysis of proton MRS studies (anterior cingulate glutathione) | Mixed; schizophrenia and bipolar disorder | Mixed; predominantly medicated | Central (MRS) | Reduced anterior cingulate glutathione in schizophrenia relative to controls | Modest pooled effect; heterogeneity in field strength, editing sequence and quantification; overlap with bipolar disorder limits disorder specificity |
| Zhang K. et al., 2025 | Narrative review | All stages | Mixed | Central and peripheral | Reduced medial prefrontal glutathione in unmedicated first-episode patients (reported figure ~52%) | Secondary source; the quantitative estimate derives from cited primary studies and is not itself a pooled estimate. Cited here as review-level synthesis |
| Cabungcal et al., 2013 | Experimental; Gclm knockout mouse with timed oxidative challenge | Preclinical; preweaning, peripubertal and young-adult windows | Not applicable; N-acetylcysteine used as intervention | Central (histology) | Glutathione deficit delays parvalbumin interneuron and perineuronal net maturation. Challenge during preweaning or puberty, but not young adulthood, reduces PV interneuron number; prevented by N-acetylcysteine | Animal model; extrapolation to human developmental timing is inferential. Strongest available directional evidence in the framework |
| Steullet et al., 2017 | Synthesis across multiple animal models | Preclinical | Not applicable | Central | Oxidative stress–driven parvalbumin interneuron impairment converges across genetic, environmental and pharmacological models | Convergence across models is suggestive of a common pathway but does not establish primacy in human disease |
| **B. Redox status — peripheral measures** | | | | | | |
| Flatow et al., 2013 | Meta-analysis | First-episode and chronic | Mixed; includes antipsychotic-naïve subgroup | Peripheral (serum, erythrocyte) | Elevated lipid peroxidation and reduced total antioxidant status; some markers behave as state variables, others as trait variables | Assay heterogeneity across studies; the state/trait distinction rests on indirect comparison rather than within-subject longitudinal data |
| Ben Othmen et al., 2008 | Case-control | Chronic | Medicated | Peripheral (plasma, erythrocyte) | Elevated malondialdehyde; altered superoxide dismutase and glutathione peroxidase activity | Cross-sectional; antipsychotic exposure not disentangled from illness; single-site sample |
| Kulaksizoglu and Kulaksizoglu, 2016 | Case-control | Chronic | Medicated | Peripheral (serum) | Neutrophil–lymphocyte ratio correlates with oxidative stress indices and psychopathology | Cross-sectional; NLR is a non-specific inflammatory index confounded by smoking, BMI and infection |
| Juchnowicz et al., 2021 | Case-control with discriminant panel construction | First-episode and chronic | Mixed | Peripheral (serum) | Total oxidant status combined with glutathione peroxidase discriminated patients from controls at approximately 89% accuracy | Healthy controls only; no psychiatric comparison group. Panel derived and evaluated in the same sample without external validation, so accuracy is optimistic |
| Mohib et al., 2025 | Cross-species: human case-control plus re-analysis of single-cell RNA-seq (Setd1a+/− mouse) | First-episode (human); preclinical (mouse) | Antipsychotic-naïve (human) | Peripheral (human serum); central (mouse prefrontal cortex) | Elevated serum malondialdehyde and nitric oxide with reduced catalase; reduced Gpx4 and Nfe2l2 (Nrf2) expression in mouse prefrontal cortex | Small human sample; the mouse model captures one rare genetic route to schizophrenia risk and may not generalize to idiopathic illness |
| Li et al., 2026 | Case-control (42 patients, 43 controls) with in vitro validation in an H₂O₂-treated neuronal model | Chronic | Medicated | Peripheral (leukocytes); central (in vitro neuronal model) | Mitochondrial fragmentation and reduced mtDNA copy number with greater oxidative damage; reduced coenzyme Q10 and SOD activity, SOD decline tracking mtDNA depletion. In vitro oxidative challenge reproduced the mitochondrial quality-control disruption | Small clinical sample; leukocytes are an imperfect proxy for neural tissue. The in vitro arm demonstrates directionality in cells, not in patients |
| Flores et al., 2026 | PRISMA systematic review of 22 observational studies (4 addressing oxidative stress) | Acute versus remitted | Mixed | Peripheral | Superoxide dismutase and total antioxidant capacity higher in remission than in acute phases; advanced oxidation protein products reduced. Erythrocyte SOD remained altered irrespective of clinical state | Constituent studies are observational and mostly cross-sectional; remission defined inconsistently across studies |
| Zhang X. et al., 2026 | Cross-sectional (n = 521) | First-episode | Drug-naïve | Peripheral (serum) | Higher serum uric acid associated with poorer immediate memory and lower global cognition — the opposite of the direction expected for an endogenous antioxidant | Cross-sectional; direction of causality undetermined. Reported here as a finding contrary to the simple antioxidant-protection account |
| Hu et al., 2026 | Case-control (89 patients, 90 controls) | Chronic | Medicated | Peripheral (serum) | Reduced SOD, catalase and glutathione peroxidase; elevated malondialdehyde; enzyme activity correlated inversely with symptom severity and metacognitive impairment | Cross-sectional; medication effects not modeled; correlational associations do not establish temporal order |
| Messamore et al., 2010; Yao et al., 2016 | Case-control; challenge test with laser-Doppler quantification | Chronic; familial samples | Mixed | Peripheral (functional skin response) | Blunted niacin skin-flush response in a patient subgroup; more prevalent in schizophrenia than in bipolar disorder or major depression; heritable within families | Sensitivity limited and method-dependent; identifies a membrane-lipid subgroup rather than functioning as a universal test |
| **C. Genetic evidence** | | | | | | |
| Tosic et al., 2006 | Case-control genetic association | Chronic | Not applicable | Genotype | GCLM associated with schizophrenia susceptibility | Candidate-gene design; does not reach genome-wide significance in subsequent large-scale GWAS. Should be presented as a mechanistically motivated candidate, not an established risk locus |
| Gysin et al., 2007 | Case-control genetic association with fibroblast functional assay | Chronic | Mixed | Genotype and peripheral (fibroblast) | GCLC trinucleotide repeat associated with schizophrenia and with lower glutamate-cysteine ligase activity and reduced glutathione | Small sample; single population; functional assay in fibroblasts rather than neural tissue. Not genome-wide significant in later GWAS |
| Trubetskoy et al., 2022 | Genome-wide association study | All stages | Not applicable | Genotype | Common-variant architecture implicating synaptic biology; redox candidate genes do not emerge at genome-wide significance | Included as a constraint on the framework rather than as support. Polygenic signal is distributed and does not privilege the redox pathway |
| Marshall et al., 2017 | Genome-wide CNV study (41,321 subjects) | All stages | Not applicable | Genotype | Rare high-penetrance copy-number variants confer substantial risk in a small minority | Explains a small fraction of cases; informative for stratification rather than for population-level mechanism |
| **D. Imaging** | | | | | | |
| Kambeitz et al., 2015 | Meta-analysis of multivariate pattern-recognition studies (38 studies; 1,602 patients, 1,637 controls) | Mixed | Mixed | Central (structural and functional MRI) | Pooled sensitivity and specificity approximately 80% for case-control classification | Case-control discrimination, not prospective prediction. Publication bias and absence of external validation inflate reported accuracy |
| Di Camillo et al., 2024 | Meta-analysis of machine-learning classification across sMRI, rs-fMRI, task fMRI and DTI | Mixed; includes CHR samples | Mixed | Central (MRI) | Approximately 80% accuracy ceiling reproduced; CHR transition prediction 70–85%. Illness stage and antipsychotic exposure identified as principal moderators | The identification of antipsychotic exposure as a moderator is itself a caution: imaging differences partly index treatment, not only illness |
| Lopes et al., 2024 | Systematic review and quantitative synthesis of proton MRS (92 studies; 2,822 patients, 2,721 controls) | Spectrum; mixed stages | Mixed | Central (MRS) | Elevated glutamate in basal ganglia, frontal and medial prefrontal cortex | Substantial between-study heterogeneity; glutamate and glutamine are difficult to resolve at standard field strengths |
| Leung et al., 2011 | Anatomical likelihood estimation meta-analysis, before and after treatment | First-episode | Compared pre- and post-antipsychotic | Central (structural MRI) | Gray-matter differences present before treatment and altered after it | Ecological comparison across studies rather than within-subject; treatment and illness progression are not fully separable |
| Komatsu et al., 2024 | Systematic review and meta-analysis of OCT and electroretinography (65 studies) | Spectrum; mixed stages | Mixed | Central (retina) | Thinning of peripapillary retinal nerve fiber layer and macular ganglion-cell layers; retinal thinning associated with illness duration and symptom severity | Cross-sectional; confounded by diabetes, hypertension and smoking, all over-represented in this population. Association with duration is not evidence of progression |
| Wengler et al., 2021 | Methodological; cross-scanner harmonization | Not applicable | Not applicable | Central (neuromelanin-sensitive MRI) | Establishes multisite comparability of the measure | Methods paper; cited for feasibility, not for a disease finding |
| Howes and Kapur, 2009; McCutcheon et al., 2019 | Reviews synthesizing PET imaging evidence | Mixed; includes CHR | Mixed | Central ([18F]-DOPA PET) | Elevated striatal presynaptic dopamine synthesis capacity, present before onset in at-risk samples | Review-level. The finding is among the more disorder-specific in the field but the underlying primary studies use small samples |
| **E. Electrophysiology** | | | | | | |
| Erickson et al., 2016 | Meta-analysis of mismatch negativity | Clinical risk through chronic illness | Mixed | Central (EEG) | Reduced MMN amplitude with the largest effect size among ERP candidates; deficits smaller in bipolar disorder than in schizophrenia; magnitude increases across stages | Paradigm heterogeneity across studies; the cross-stage gradient is assembled from separate cohorts rather than followed within individuals |
| Hamilton et al., 2022 | Prospective longitudinal cohort | Clinical high risk | Predominantly unmedicated | Central (EEG) | MMN amplitude deficit associated with subsequent transition to psychosis | Association modest; limited predictive value as a standalone measure. This is one of the few genuinely prospective datasets available to the framework |
| Perrottelli et al., 2021 | Systematic review of EEG measures | At-risk and early illness | Mixed | Central (EEG) | Reduced MMN and P300 amplitude and impaired sensory gating are the most consistent abnormalities | Narrative synthesis without pooled estimates; protocols not standardized across sites |
| Tada et al., 2025 | Review | Mixed | Mixed | Central (EEG) | Reviews translational status of MMN and its route to real-world application | Review-level; cited for translational framing rather than as primary evidence |
| **F. Inflammation and kynurenine pathway** | | | | | | |
| Goldsmith et al., 2016 | Meta-analysis | Acute relapse, first-episode and chronic | Mixed | Peripheral (serum, plasma) | IL-6, TNF-α, IL-1β, IL-8 and IFN-γ elevated, most markedly in first episode; some cytokines behave as state markers, others as trait markers | Substantial overlap with mood disorders; BMI, smoking and metabolic status incompletely controlled across constituent studies |
| Khandaker et al., 2015 | Review incorporating longitudinal birth-cohort data | Premorbid through established illness | Predominantly untreated at exposure measurement | Peripheral | Childhood inflammatory markers associated with later psychotic experiences | Observational; residual confounding by early adversity and infection burden cannot be excluded |
| Fillman et al., 2016 | Case-control with unsupervised subgrouping | Chronic | Medicated | Peripheral and post-mortem cortical | A high-cytokine subgroup is identifiable within schizophrenia and differs in cortical and clinical profile | Subgroup boundaries are analytically derived and not yet independently replicated with the same thresholds |
| Chen et al., 2022 | Case-control panel construction | First-episode | Largely untreated | Peripheral (serum) | Endotoxin, HMGB1 and complement C4 combined discriminated first-episode schizophrenia from controls with AUC 0.933 | Discovery cohort only; healthy controls as sole comparator, so specificity against other psychotic disorders is untested |
| Marx et al., 2021 | Meta-analysis of 101 studies across MDD, bipolar disorder and schizophrenia | Mixed | Mixed | Peripheral and CSF | Kynurenine pathway dysregulation confirmed in schizophrenia, with reduced plasma kynurenine | Transdiagnostic design demonstrates that the abnormality is shared rather than schizophrenia-specific |
| Cao et al., 2021; Plitman et al., 2017 | Meta-analyses | Mixed | Mixed | CSF and peripheral | Elevated CSF kynurenic acid replicated in schizophrenia | Shared with bipolar disorder; CSF sampling restricts sample sizes and introduces selection effects |
| **G. Neurotrophic, metabolomic and proteomic markers** | | | | | | |
| Fernandes et al., 2015 | Meta-analysis | Mixed | Explicitly modeled antipsychotic effect | Peripheral (serum, plasma) | Peripheral BDNF reduced (effect size approximately −0.5 to −0.7); levels change with antipsychotic treatment | Peripheral BDNF correlates only weakly with central concentrations; treatment sensitivity limits its use as a trait marker |
| Green et al., 2011; Ahmed et al., 2021 | Meta-analyses | First-episode and chronic | Mixed | Peripheral | Reduced BDNF replicated across independent syntheses | Assay platform heterogeneity; serum and plasma values are not interchangeable |
| Dombi et al., 2022 | Review | Spectrum | Mixed | Peripheral | BDNF functions as an index of neuroplastic compromise across the schizophrenia–bipolar spectrum | Explicitly transdiagnostic; cited as a constraint on specificity claims |
| Davison et al., 2018; Yao G. et al., 2025 | Systematic reviews of metabolomic studies | Predominantly onset and early illness | Mixed | Peripheral (serum, plasma, urine) | Reproducible shifts in lipid, amino-acid and energy metabolites, several redox- and glutamate-related | Few studies use common platforms or normalization; diet, fasting state and medication strongly influence the metabolome |
| Zhang Y. et al., 2024 | Discovery proteomics (4D-DIA) | Mixed | Mixed | Peripheral (plasma) | Protein classification models reached AUC above 0.97 | Discovery cohort without independent validation; performance at this level in small samples is characteristically optimistic |
| Zaki et al., 2022 | Case-control; PBMC subtype-specific expression modeling | Mixed | Mixed | Peripheral (PBMC) | Metabolic marker expression in specific immune-cell subsets supports diagnostic modeling | Single cohort; cell-subtype resolution improves plausibility but not external validity |
| **H. Gut microbiome** | | | | | | |
| Zhu et al., 2020 | Metagenome-wide association study | Mixed | Medicated | Peripheral (faecal) | Reproducible compositional differences, Streptococcus repeatedly implicated; altered short-chain fatty acid, tryptophan and GABA/glutamate synthesis modules | Antipsychotics alter the microbiome directly; diet is a major uncontrolled confounder |
| Huang et al., 2026 | Integrated metagenomic and metabolomic modeling | Mixed | Mixed | Peripheral (faecal, serum) | Combined models discriminated patients from controls with AUC up to 0.94 | Discovery cohort; unstandardized assays; no individual-level interpretive framework exists |
| Zheng et al., 2019 | Experimental; faecal microbiota transfer into germ-free mice | Preclinical | Not applicable | Central (mouse brain) following peripheral transfer | Transfer from patients induced schizophrenia-relevant behavioral and glutamate–glutamine–GABA cycle changes, indicating causal contribution rather than association alone | Germ-free mice are a highly artificial host; behavioral readouts have limited construct validity for psychosis |

*Abbreviations: AUC, area under the receiver operating characteristic curve; BDNF, brain-derived neurotrophic factor; BMI, body mass index; CHR, clinical high risk; CNV, copy-number variant; CSF, cerebrospinal fluid; DTI, diffusion tensor imaging; EEG, electroencephalography; ERP, event-related potential; GCLC/GCLM, catalytic and modifier subunits of glutamate-cysteine ligase; GWAS, genome-wide association study; HMGB1, high-mobility group box 1; IFN, interferon; IL, interleukin; MDD, major depressive disorder; MMN, mismatch negativity; MRS, magnetic resonance spectroscopy; mtDNA, mitochondrial DNA; NLR, neutrophil–lymphocyte ratio; OCT, optical coherence tomography; PBMC, peripheral blood mononuclear cell; PET, positron emission tomography; rs-fMRI, resting-state functional magnetic resonance imaging; SOD, superoxide dismutase; sMRI, structural magnetic resonance imaging; TNF, tumor necrosis factor.*
