# Supplementary material for: Redox compromise as an early, modifiable entry point in schizophrenia: a staging framework for early intervention
Source: Front Neurosci. 2026 Oct 15;20:1941148. doi: 10.3389/fnins.2026.1941148 (PMC13716746; doi:10.3389/fnins.2026.1941148)
Supplement: Supplementary file 4 [file Data_Sheet_4.DOCX]

**Supplementary Statistical Note**

The reasoning behind the numbers in Sections 3.6 and 6.4

Leonard Lado and Aruna Misir

# How to read this note

This note is written for two audiences.

**Part A is for clinicians.** It explains, for psychiatrists and other physicians, what the numbers in the manuscript mean and why they matter, using the same reasoning that underlies everyday diagnostic testing: pretest probability, how tests combine, and what a result means for one patient. No statistical training is needed, and readers who want the reasoning can stop there.

**Part B is for researchers designing studies.** It gives the formal derivations, so that a statistician can check every figure, and it is built to be reused. Section B4 lists every input on which the calculations depend. A research group planning its own study can replace the inputs with values from its own population, such as its conversion rate, the performance of its own assays, or the variance measured in a pilot, and rerun the calculation in statistical software or with the help of an AI assistant. Calculations used to plan a study that will actually enroll participants should be confirmed by a statistician.

All the numbers are illustrations built on stated assumptions. None comes from patient data.

# Part A: The reasoning in clinical terms

## A1. How much does a panel of three tests change a patient’s risk?

Start with a group of 100 young people referred to a clinical high risk service. On current evidence, about 25 of them will develop psychosis within three years. That is the pretest probability, one in four.

Now suppose each person has three biomarker tests, one for inflammation, one for redox status and one for brain circuit function, and suppose each abnormal result makes transition somewhat more likely. The question is what a patient’s risk becomes when all three come back abnormal.

The answer depends on one thing clinicians already know from ordinary practice: **whether the tests tell you different things or the same thing.** A troponin adds a great deal to an ECG because the two measure different aspects of cardiac injury. A second troponin drawn five minutes after the first adds almost nothing, because it repeats information already in hand. Tests only add up when they are independent.

If the three biomarkers were fully independent, the arithmetic is straightforward:

| Result | Out of 100 people with this result, how many develop psychosis |
| --- | --- |
| No testing (starting point) | 25 |
| One test abnormal | about 43 |
| All three abnormal | about 80 |
| All three normal | about 6 |

That is the most optimistic case. The difficulty is that this manuscript itself argues that inflammation, redox imbalance and circuit dysfunction are parts of one interconnected process. If that is true, the three tests partly measure the same thing, like two troponins, and they add less than the table suggests. The manuscript cannot claim both, and the table below takes the overlap into account.

So the realistic answer is a range, depending on how much the tests overlap:

| How much the three tests overlap | All three abnormal | All three normal |
| --- | --- | --- |
| Not at all (fully independent) | about 80 of 100 | about 6 of 100 |
| Partly (worth about two independent tests) | about 64 of 100 | about 10 of 100 |
| Completely (worth only one test) | about 43 of 100 | about 16 of 100 |

**The clinical message.** Even in the best case, a fully abnormal panel is wrong about one person in five, and a young person flagged in error faces years of monitoring for an illness that never comes. A normal panel is more useful: it lowers risk to somewhere between one in six and one in sixteen, which could reasonably support easing monitoring. Where a real panel falls in this range can only be learned by measuring all three tests in the same people and seeing how closely they track each other.

## A2. Why glutathione alone cannot classify anyone

Medial prefrontal glutathione on MR spectroscopy is the most direct measure of the biology this manuscript proposes. It is lower in patients than in controls on average. The question is whether it is useful for one patient.

The pooled difference between patients and controls is small. A practical way to picture it: **pick one patient and one healthy person at random. The patient has the lower glutathione level only about 56 times in 100.** A coin toss would get it right 50 times in 100.

Put another way, an abnormal glutathione result raises the odds of illness by a factor of about 1.2. For the panel arithmetic in A1 to work, each test would need to raise the odds by about 2.3, which requires a difference between patients and controls roughly five times larger than glutathione shows.

**The clinical message.** Glutathione is a meaningful group finding and a poor individual test. It can contribute to a panel. It cannot define a subtype on its own, which is why no rule in this framework allows a single glutathione value to assign a redox subtype.

## A3. How many patients would the proposed trial need?

The trial asks a specific question. Not simply “does an antioxidant help?” but **“does it help people with the redox subtype more than it helps everyone else?”** That second question is harder to answer, for the same reason a subgroup finding in any trial needs more patients: it compares two treatment effects rather than measuring one. As a rule of thumb, it needs about four times as many patients as the simple question.

**If the outcome is conversion to psychosis,** the numbers become very large, because conversion is uncommon. Assuming the treatment halves conversion in the redox subtype (from 20 in 100 to 10 in 100) and does nothing for others, the trial needs about 1,400 participants. That is not a realistic first trial.

**If the outcome is a brain measurement,** the change in glutathione on MR spectroscopy, the trial becomes feasible. Every participant contributes a measurement, rather than only the minority who convert, so far fewer are needed: about 600, allowing for dropouts. Conversion is still recorded, as a secondary outcome.

**The clinical message.** The first trial should test whether treatment changes the biology in the people the framework says it should. Only if it does is the much larger trial of conversion to psychosis worth running. A negative first trial would count against the framework at a fraction of the cost.

## A4. What these numbers are not

They are not estimates. They rest on assumptions: that one in four converts, that each test raises risk by a particular amount, and that treatment has an effect of a particular size. Each assumption is stated in Part B, and each should be replaced with measured values as soon as data exist.

# Part B: Formal derivations

## B1. Combining test results

Risk is updated on the odds scale. For a prior probability *p*, the prior odds are *p* / (1 − *p*). Each result multiplies the odds by its likelihood ratio: LR+ for an abnormal result, LR− for a normal one. Posterior probability is odds / (1 + odds).

The prior is 0.25, close to the pooled three-year cumulative risk reported by Salazar de Pablo et al. (2021), giving prior odds of 1/3.

LR+ is set at 2.3 for illustration. LR− is chosen so that three normal results under independence give a posterior of 1/16:

(1/3) × LR−³ = (1/16) / (15/16) = 0.0667, so LR−³ = 0.200 and LR− = 0.585.

Under conditional independence the three likelihood ratios multiply. To bracket the effect of positive correlation between markers, three markers are treated as carrying the information of *k* independent markers, with *k* from 1 (fully redundant) to 3 (independent); posterior odds are (1/3) × LR^*k*. This is a bracketing device rather than a fitted dependence model.

| *k* | All three abnormal | All three normal |
| --- | --- | --- |
| 3 | 0.80 | 0.063 |
| 2 | 0.64 | 0.10 |
| 1 | 0.43 | 0.16 |

One abnormal marker alone: (1/3) × 2.3 = 0.767, probability 0.43.

## B2. From effect size to test performance

Assume a binormal model with equal variances, standardized group difference *d*, and a cutoff midway between group means, so sensitivity equals specificity. Then sensitivity = specificity = Φ(*d*/2), LR+ = Φ(*d*/2) / (1 − Φ(*d*/2)), and AUC = Φ(*d*/√2), where Φ is the standard normal distribution function.

| *d* | Sensitivity = specificity | LR+ | LR− | AUC |
| --- | --- | --- | --- | --- |
| 0.21 | 0.54 | 1.18 | 0.85 | 0.56 |
| 0.5 | 0.60 | 1.49 | 0.67 | 0.64 |
| 0.8 | 0.66 | 1.90 | 0.53 | 0.71 |
| 1.0 | 0.69 | 2.24 | 0.45 | 0.76 |
| 1.2 | 0.73 | 2.65 | 0.38 | 0.80 |

An LR+ of 2.3 requires *d* near 1.0. The pooled medial prefrontal glutathione effect, *d* = −0.21 (Sydnor and Roalf, 2020), gives LR+ of about 1.2 and AUC of about 0.56. The AUC is the probability that a randomly chosen patient has a lower value than a randomly chosen control, which is the basis of the “56 in 100” statement in A2.

## B3. Sample size for the treatment-by-subtype interaction

Two-sided α = 0.05, power 80%, so *z*₁₋α/₂ + *z*₁₋β = 1.960 + 0.842 = 2.802. The pre-specified contrast is redox subtype against all others, giving a 2 × 2 design with *m* participants per cell.

**Binary outcome (transition).** Assumed proportions: redox subtype 10% with adjunct, 20% without; others 20% in both arms. The interaction on the log-odds scale is ln[(0.10/0.90) / (0.20/0.80)] = −0.811. Its variance is (1/*m*) × Σ 1/[*p*(1 − *p*)] = (11.11 + 6.25 + 6.25 + 6.25)/*m* = 29.86/*m*. Solving 0.811 / √(29.86/*m*) = 2.802 gives *m* = 356.4, so 357 per cell and 1,428 in total. By contrast, about 600 participants spread across three subtypes and two arms gives roughly 100 per cell and 15 to 20 events per cell at a 15 to 20% transition rate.

**Continuous outcome (change in glutathione).** With standardized interaction δ, each within-subtype treatment effect has variance 2σ²/*m*, so the interaction has variance 4σ²/*m*. Solving δ√*m* / 2 = 2.802 with δ = 0.5 gives *m* = 125.6, so 126 per cell, 504 in total, 593 allowing for 15% attrition. A main effect of the same size in a two-arm trial needs 63 per arm, 126 in total; the interaction needs four times that.

δ = 0.5 is an assumption. The variance of within-person change in spectroscopic glutathione and its test-retest reliability are not established, and the calculation should be repeated once pilot data supply them. Covariate adjustment would be expected to reduce the required sample and has not been assumed.

## B4. Adapting the calculations to a new study

Every number in this note follows from the inputs below and the formulas in Sections B1 to B3. To adapt the calculations to a new study, replace the inputs with values from that study and recompute.

| Input | Value used here | What to replace it with |
| --- | --- | --- |
| Pretest probability of conversion | 0.25 | Conversion rate in your referral population |
| Odds multiplier, one abnormal marker | 2.3 | Likelihood ratio of your assay, from validation data |
| Risk after three normal markers | 1/16 | Or set the normal-result likelihood ratio directly |
| Effect size of a single marker | 0.21 | Standardized difference for your marker |
| Significance level and power | 0.05 and 0.80 | Your trial’s chosen values |
| Conversion rates in each subtype and arm | 0.10, 0.20, 0.20, 0.20 | Your expected rates |
| Standardized interaction, continuous outcome | 0.5 | Estimate from pilot data |
| Attrition | 0.15 | Expected dropout in your setting |

A program implementing these calculations is available from the corresponding author on request.

# References

Salazar de Pablo, G., Radua, J., Pereira, J., Bonoldi, I., Arienti, V., Besana, F., et al. (2021). Probability of transition to psychosis in individuals at clinical high risk: an updated meta-analysis. *JAMA Psychiatry* 78, 970–978. doi:10.1001/jamapsychiatry.2021.0830

Sydnor, V. J., and Roalf, D. R. (2020). A meta-analysis of ultra-high field glutamate, glutamine, GABA and glutathione 1H-MRS in psychosis: implications for studies of psychosis risk. *Schizophr. Res.* 226, 61–69. doi:10.1016/j.schres.2020.06.028
